# Soft Ferroelectrics in One Dimension: Ferroelectric Columnar Liquid Crystals

**DOI:** 10.1002/cplu.70185

**Published:** 2026-06-09

**Authors:** Seongwon Park, Byoung‐Ki Cho

**Affiliations:** ^1^ Department of Chemistry Dankook University Chungnam Korea

**Keywords:** axial polarization, ferroelectric, helical columnar, liquid crystal, memory device

## Abstract

Ferroelectric columnar liquid crystals (FCLCs) are attractive candidates for ultra‐high‐density organic memories because bistable information can be encoded within individual polar columns. However, most “switching” columnar LCs relax into paraelectric or antiferroelectric states once the electric field is removed, which severely limits their practical utility. In this concept article, we summarize the limited number of FCLC systems reported to date and present a materials‐design perspective that integrates molecular design with supramolecular locking/structural stabilization strategies to achieve long‐lived axial polarization. Central to this framework is suppressing rotation‐driven depolarization by “locking” polar motifs through directional hydrogen‐bonding and steric confinement, which is often realized as well‐defined intracolumnar helical order. In particular, we discuss how representative polar building blocks—amide, thioamide, benzene‐1,3,5‐tricarboxamide (BTA), urea, and the recently emerging aromatic 1,2,3‐triazole unit—govern polar switching and polarization stabilization. Finally, we propose actionable molecular and device‐level strategies to further enhance FCLC performance and accelerate their translation toward memory applications.

## Graphical abstract

Helical ferroelectric columnar liquid crystals (FCLCs) confine bistable axial polarization within individual columns. An electric field reversibly flips the macrodipole, enabling two nonvolatile states readable as “0” and “1.” This Cconcept highlights supramolecular locking and helical architectures that deliver genuine switching and long‐lived zero‐field retention.

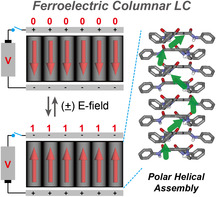

## 1 Introduction

**FIGURE 1:**
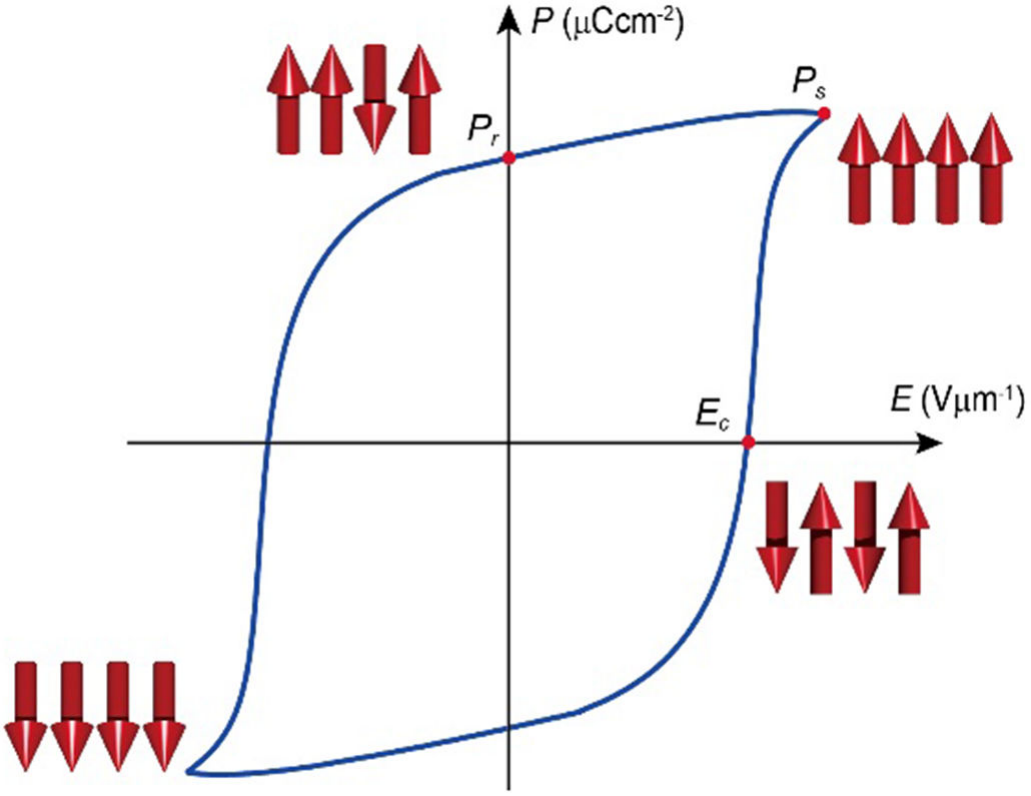
Schematic *P–E* hysteresis loop of a ferroelectric material. The loop highlights the *P*_r_ retained at zero E‐field and the coercive field (*E*_*c*_) required to reverse the polarization. *P*_s_ denotes the spontaneous polarization, and the inset cartoons illustrate the corresponding dipole configurations.

Ferroelectric materials exhibit a reversible inversion of macroscopic polarization under an external electric field (E‐field), producing a characteristic hysteresis loop (Figure 1). Because the polarization does not vanish completely when the field is removed but partially remains as a remnant polarization (*P*_r_), ferroelectrics can store binary information (0/1) and ideal candidates for data‐storage applications such as ferroelectric random‐access memory (FeRAM) [1] and radio‐frequency identification (RFID) devices [2].

Ferroelectricity is also intrinsically linked to pyroelectric and piezoelectric responses, enabling broader implementation in sensor platforms, including infrared detectors [3], actuators [4], temperature sensors [5], and transducers [6]. In practice, industrial technologies continue to depend largely on oxide‐based inorganic ferroelectrics, including lead zirconate titanate (PZT) [7], barium titanate (BaTiO_3_) [8], and lead titanate (PbTiO_3_) [9], which can suffer from drawbacks such as toxicity, high density, high‐temperature processing requirements, and elevated fabrication costs. In this context, organic ferroelectrics have emerged as attractive alternatives owing to their low‐temperature processability, light weight, and mechanical compliance [10, 11].

**FIGURE 2:**
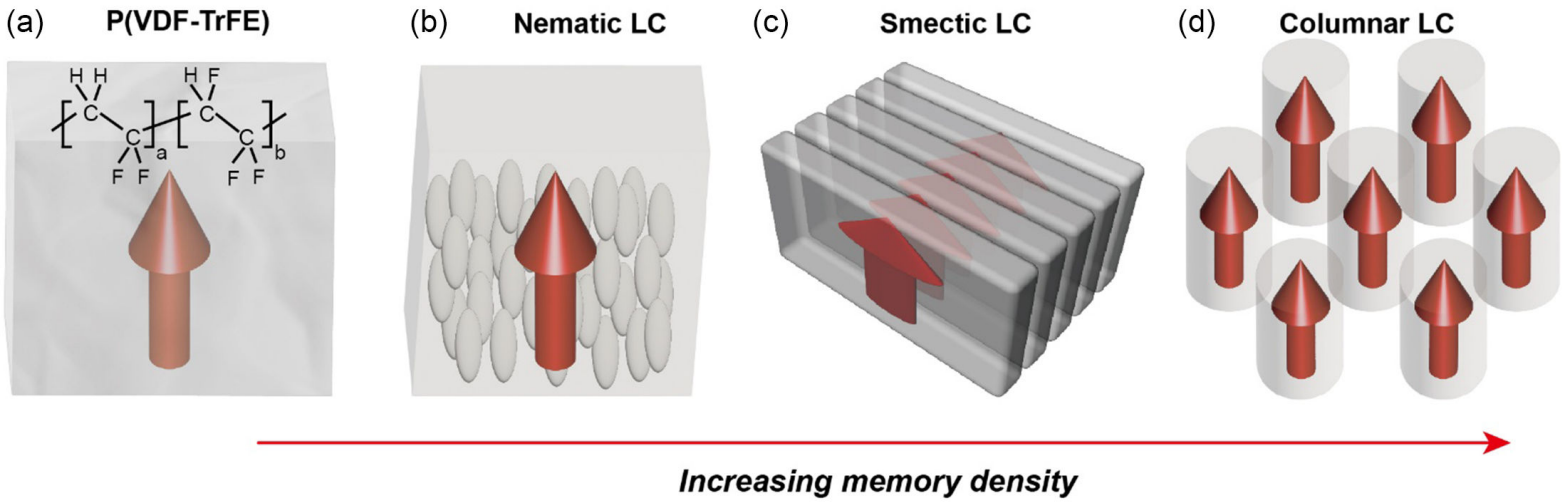
Schematic comparison of polarization domain dimensionality and addressable domain density in representative soft ferroelectrics. (a) Ferroelectric polymers (e.g., P(VDF–TrFE)), where aligned dipoles generate a continuous bulk polarization, typically forming a single macroscopic polarization domain over a given area without discrete nanoscale domain partitioning. (b) Ferroelectric nematic LCs, in which spontaneous polarization develops along the nematic director in a fluid and orientationally ordered phase, while the number of individually addressable polarization domains per given area remains limited compared with more spatially confined LC architectures. (c) Smectic LCs, in which polarization can also be confined to individually addressable layered domains. (d) CLCs, where axial macrodipoles are confined within discrete 1D columns, yielding a higher areal density of individually addressable polarization domains (red arrows indicate the polarization direction.).

Among organic ferroelectrics, ferroelectric polymers such as poly(vinylidene fluoride) (PVDF) and poly(vinylidene fluoride–trifluoroethylene) (P(VDF‐TrFE)) exhibit fast switching and high *P*_r_ [12]. However, for memory applications, these ferroelectric polymer films are typically continuous**,** making it nontrivial to define electrically isolated bit cells without additional microfabrication and stringent domain control (Figure 2a) [13]. Recent studies on ferroelectric nematic LCs (N_F_) have further expanded the landscape of soft ferroelectrics by demonstrating that even a fluid nematic phase can exhibit spontaneous macroscopic polar order, ferroelectric switching, giant dielectric responses, and high fluidity [14, 15]. However, from the viewpoint of information storage, ferroelectric nematics still resemble ferroelectric polymer films in an important respect: Although polar domains and domain walls may appear through director textures, the polarization is not intrinsically partitioned into structurally discrete, pre‐organized nanoscale compartments. Therefore, unlike layered smectic or columnar architectures, ferroelectric nematic LCs do not inherently provide a dense array of individually addressable polarization units within a given area (Figure 2b). In contrast, more ordered ferroelectric LCs can self‐assemble into structurally compartmentalized domains, offering a potential route to intrinsic nanoscale data‐storage units. In ferroelectric smectic LCs [16, 17], for example, each smectic layer may behave as a single polarization domain; in principle, a bit could be encoded by switching the polarization of an individual layer between 0 and 1 (Figure 2c) [18].

Building on this trajectory, columnar LCs represent a more advanced memory paradigm in terms of polarization‐domain density. If axial polarization forms along the columnar axis and can be reversibly switched, a single column could serve as a bistable memory unit. Compared with smectic LCs composed of 2D‐layered domains, columnar phases can concentrate a larger number of polarization domains, offering a theoretical advantage for ultra‐high‐density memory (Figure 2d) [19, 20]. Nevertheless, in early demonstrations of electrically responsive columnar systems, spontaneous polarization (*P*_s_) was frequently canceled at zero bias, leaving little to no macroscopic remnant polarization. Thus, even when a “switching current” was observed, most systems were classified not as genuinely ferroelectric but as relaxing into paraelectric or antiferroelectric states at zero field [21, 22, 23, 24, 25, 26]. Related columnar systems have also shown field‐responsive currents, yet assigning these signals to true ferroelectric switching has remained challenging because contributions from ionic migration and charge‐injection artifacts are difficult to exclude [27, 28].

**FIGURE 3:**
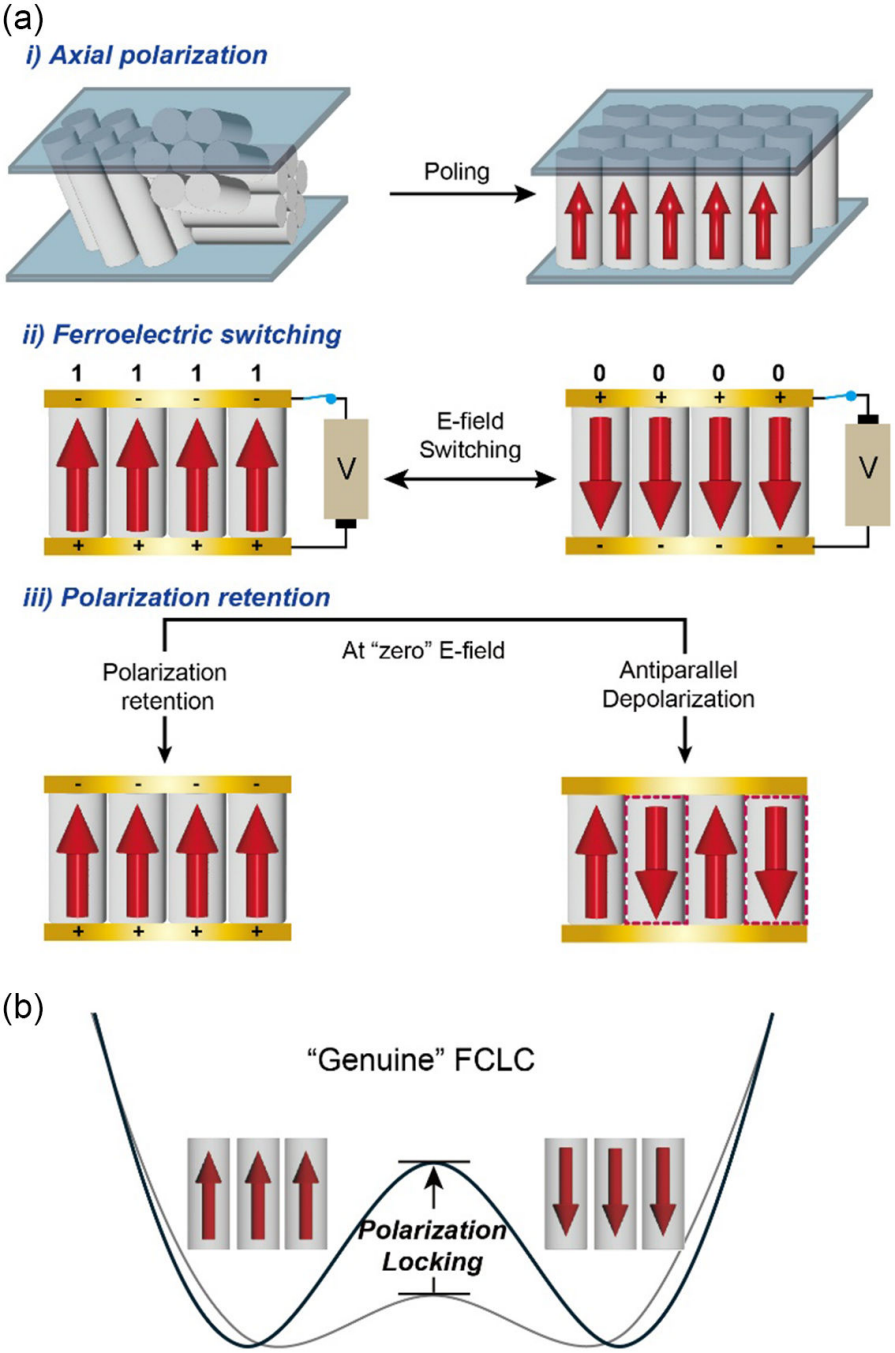
(a) Criteria for genuine FCLCs and (b) the role of polarization locking.

Therefore, realizing practical ferroelectric function in columnar LCs (CLCs) requires satisfying three criteria simultaneously: (i) generation of axial polarization, (ii) polarization switching, and (iii) retention of polarization at zero field (Figure 3a). From this perspective, the success of ferroelectric CLCs (FCLCs) cannot be reduced to a single question such as “how polar is the column.” Rather, the key challenge is to increase the energy barrier between two polar states while still enabling reversible switching under an applied field [29]. This demands precise control over molecular and supramolecular degrees of freedom. In columnar assemblies, depolarization can proceed rapidly via rotation of polar units. Therefore, hydrogen‐bonding (H‐bonding)‐mediated directional interactions or steric confinement are required to “lock” polar motifs in place (Figure 3b). This positional immobilization of polar units within the column, enabled by specific directional interactions, can be inferred from the emergence of helical columns featuring well‐defined intracolumnar order. A supramolecular‐level mechanistic understanding of polarization retention is therefore essential. Nevertheless, the potential role of helical column architectures as a structural prerequisite for “genuine” FCLC behavior has received comparatively limited discussion to date.

Accordingly, in this concept, we describe molecular design strategies for realizing genuine FCLCs. Specifically, we discuss directional H‐bonding motifs—such as amide, benzene‐1,3,5‐tricarboxamide (BTA), and thioamide and urea—along with the recently highlighted polar aromatic unit 1,2,3‐triazole, which can promote directional supramolecular interactions. Their key ferroelectric properties, including *P*_r_, operating temperature, coercive field (*E*_c_), and retention time, are summarized in Table 1. We propose that these polar units are not merely dipole providers; rather, by introducing strong intermolecular interactions (e.g., H‐bonding), they can induce supramolecular polarization locking within columns, stabilize helical column architectures, and thereby suppress rotation‐driven depolarization. Ultimately, this design framework is expected to enable “genuine” FCLC behavior that simultaneously delivers reversible polarization switching and long‐term retention.

**TABLE 1:** Summary of representative FCLC systems.

Polar group	Sample	***P***_**r,**_ **μC cm** ^**−2**^	**Operating** **temperature,°C**	***E***_**c,**_ **V μm** ^**−1**^	Retention time
Amide	**6**	1.7	120	0.23	>16 h
	**7**	2.5	RT‐130	100–300	>10 years
Benzene‐1,3,5‐tricarboxamide	**12**	6.7	RT	100	>8 days
	**13**	3.4	RT‐78	210 (at 70°C)	>10^11^ s
	**13/9**	3.3	RT‐130	100 (at 70°C)	>3 months
Thioamide	**14**	4.5	RT‐80	200 (at RT)	>10 years
Urea	**21**	0.79	110–140	4.12 (at 140°C)	>10 h
	**22**	0.79	134–144	3.6 (at 134°C)	>6 h
	**23**	0.83	125–140	6.22 (at 135°C)	>24 h
	**25**	Not specified	50	5.6 (at 50°C)	>24 h
	**26**	0.2	RT‐50	3.5 (at RT)	>2 months
Triazole	**32**	0.93	RT‐101	54 (at RT)	>8 months

## 2 Columnar Liquid Crystals: Self‐Assembly, Intracolumnar Order, and Alignment

**FIGURE 4:**
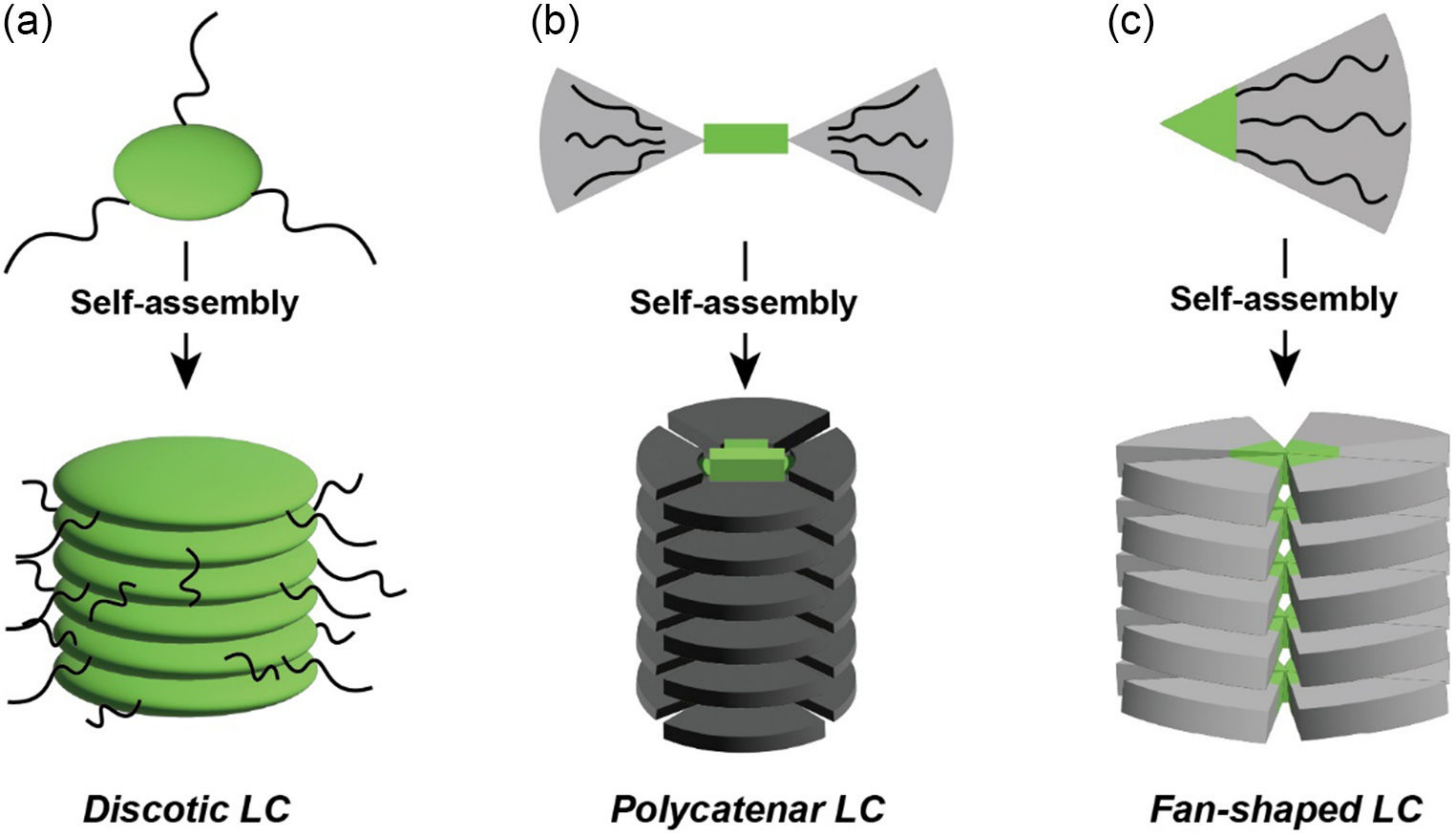
Representative molecular motifs of (a) discotic, (b) polycatenar, and (c) fan‐shaped LCs, illustrating their self‐assembly into columnar liquid crystalline architectures.

Columnar liquid crystals (CLCs) are self‐assembled mesophases in which molecules organize into one‐dimensional (1D) columns, and these columns further pack into 2D lattices. The most classical molecular motif is the discotic mesogen (Figure 4a). Discotic molecules typically feature a rigid, planar, π‐conjugated aromatic core (e.g., benzene‐, triphenylene‐, or hexabenzocoronene‐based units) decorated with multiple flexible peripheral chains such as alkyl or oligoether substituents [30, 31, 32]. When these molecules acquire sufficient mobility in the melt or within an appropriate temperature window, cooperative noncovalent interactions—including π–π stacking, dipolar interactions, and van der Waals forces—drive face‐to‐face aggregation of the disc‐like cores, generating elongated columns that extend along a single axis. In this process, the flexible chains segregate outward to fill space around the column, and microsegregation between the rigid core region and the flexible‐chain region plays a central role in stabilizing the columnar architecture.

CLCs are not limited to discotic mesogens; they are also frequently observed in polycatenar [33, 34] or fan‐shaped molecules (Figure 4b,c) [35, 36]. Polycatenar mesogens generally comprise a relatively linear or triangular/wedge‐shaped core bearing multiple flexible alkyl chains, and fan‐shaped mesogens are a wedge‐like LC molecule in which peripheral substituents radiate outward from a compact core. Here again, column formation emerges from a competition between the tendency of the cores to aggregate and the steric/entropic demands of the chains. The resulting structure often resembles a core‐rich interior surrounded by a chain‐rich exterior. Thus, columnar phases are not exclusive to “disc‐shaped” molecules; rather, they represent a general self‐assembly motif that can be realized across diverse molecular families through shape anisotropy and tailored intermolecular interactions. Once formed, columns typically arrange into 2D lattices. Among the possible lattice symmetries, the hexagonal columnar phase (Col_hex_) is by far the most widely observed. In Col_hex_, columns pack with sixfold symmetry and often achieve the most efficient areal packing. Experimentally, small‐ and wide‐angle X‐ray scattering (SAXS/WAXS) provides diagnostic peak‐position ratios characteristic of hexagonal packing (e.g., 1:√3:2:√7, etc.), enabling reliable phase identification and lattice‐parameter extraction. Polarized optical microscopy (POM) can reveal characteristic fan‐ or mosaic‐like textures for columnar mesophases [37], and differential scanning calorimetry (DSC) is commonly used to delineate thermal stability windows through crystal–mesophase–isotropic transitions.

**FIGURE 5:**
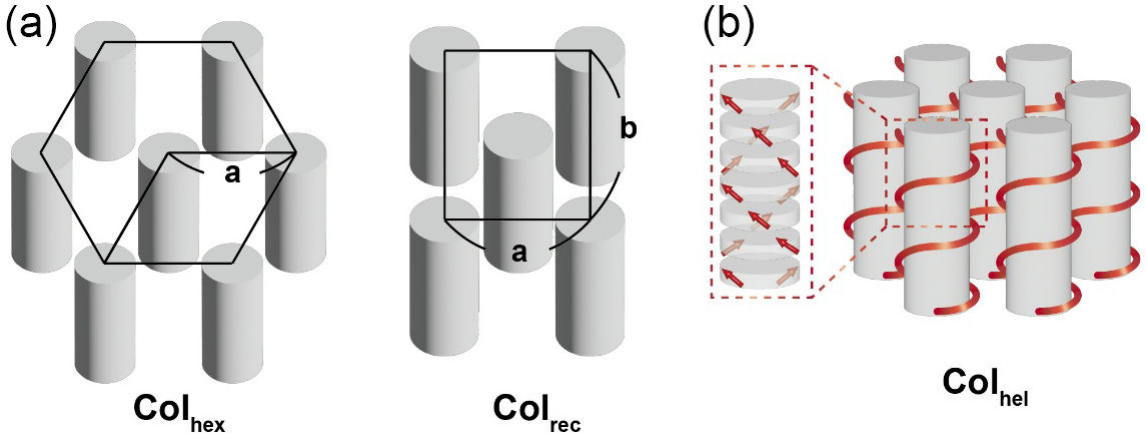
Schematic illustration of representative columnar structures. (a) Two‐dimensional column lattices: hexagonal columnar (Col_hex_) and rectangular columnar (Col_rec_) arrangements, with the lattice parameters **a** and **b** indicated. (b) Helical columnar phase (Col_hel_), in which the intracolumnar stacking adopts a helical order (inset).

A particularly important feature of CLCs is that a “column” is not merely a stack of molecules, but can exhibit widely varying degrees of intracolumnar order. In some systems, discotic cores maintain relatively regular stacking distances and display quasi‐crystalline order along the column axis; in others, the column retains a more liquid‐like character with significant axial fluctuations. Because intracolumnar order directly impacts functional properties—such as charge transport, ion mobility, and polarization retention—it is often insufficient to describe a material solely by its 2D lattice symmetry (Col_hex_ vs. Col_rec_) (Figure 5a). Instead, one must also consider how well molecules are organized within each column. Moreover, when chirality is present or when specific directional interactions (e.g., H‐bonding, amide/urea motifs, or strong dipolar alignment) dominate, molecules can adopt a progressively rotating arrangement along the column axis, giving rise to helical CLC phase (Col_hel_) (Figure 5b). Such helical order can serve as an important structural clue when discussing ferroelectricity and polarization stabilization in columnar assemblies.

The driving forces for columnar phase formation can be rationalized as an enthalpy‐entropy balance; attractive interactions favor columnar order, but overly rigid locking of molecular positions reduces the configurational freedom of flexible chains, increasing entropic cost. Consequently, subtle variations in side‐chain length, number, and branching, as well as core size, planarity, and the introduction of polar groups, can strongly influence phase stability, transition temperatures, lattice symmetry, and defect density. For instance, very short chains can favor crystallization, whereas overly long chains may dilute core–core interactions and promote isotropization; intermediate chain designs often stabilize broad Col_hex_ windows.

CLCs are intrinsically anisotropic, and their properties depend strongly on orientation [30]. In practical applications, if the columns are randomly oriented or fragmented into polydomains, the anisotropy‐derived advantages are averaged out and thus diminished, making alignment a critical processing step. Depending on the material, alignment can be induced by shear in the melt [38], solvent/thermal annealing in thin films to promote domain growth [39, 40, 41], surface‐alignment strategies (alignment layers, SAMs, and rubbing) [42, 43, 44], or the application of external electric or magnetic fields [45, 46].

Columnar orientation is also crucial for FCLCs. In FCLCs, E‐field‐assisted alignment can, in principle, be combined with poling to orient macrodipoles parallel to the applied field and to promote more uniform polarization domains across the 2D plane. Achieving such controlled orientation and homogeneous domain formation is therefore a key processing requirement for memory‐device implementations.

In summary, discotic, polycatenar, and fan‐shaped molecules self‐assemble into 1D columns that pack into hexagonal or rectangular lattices. Core–chain segregation, π–π stacking, specific directional interactions, and alignment/defect control collectively govern their structures and properties. In particular, intracolumnar order—including the possibility of helical organization—provides a key structural handle for advancing functional performance, spanning charge transport, and polarization stabilization in next‐generation FCLCs.

## 3 E‐Field‐Responsive CLCs with Amide Linkages

Amides, owing to their chemical and thermal stability, strong H‐bonding capability, and a permanent dipole moment of ∼3 Debye [47, 48], have served as useful polar motifs for designing polar CLC materials. In CLC systems, intermolecular H‐bonding between amide groups not only stabilizes mesogenic orientation but also contributes to the formation of a columnar macrodipole. For these reasons, many of the polar CLC studies reported to date have focused on amides and their derivatives [49, 50, 51, 52, 53]. In particular, efforts to realize invertible columnar macrodipoles have emphasized both the H‐bonding network and the rotational/orientational dynamics of the amide functionalities.

In the following section, amide‐based E‐field‐responsive CLCs and FCLCs are discussed. Although numerous amide‐containing polar CLCs have been reported, examples that simultaneously satisfy polar switching and polarization retention remain limited. Therefore, this section focuses on early amide‐based CLCs that respond to an applied electric field and on the molecular design strategies developed by the Aida group to realize genuine FCLCs.

### 3.1 E‐Field‐Induced Orientation of CLCs With Amide Linkages

**FIGURE 6:**
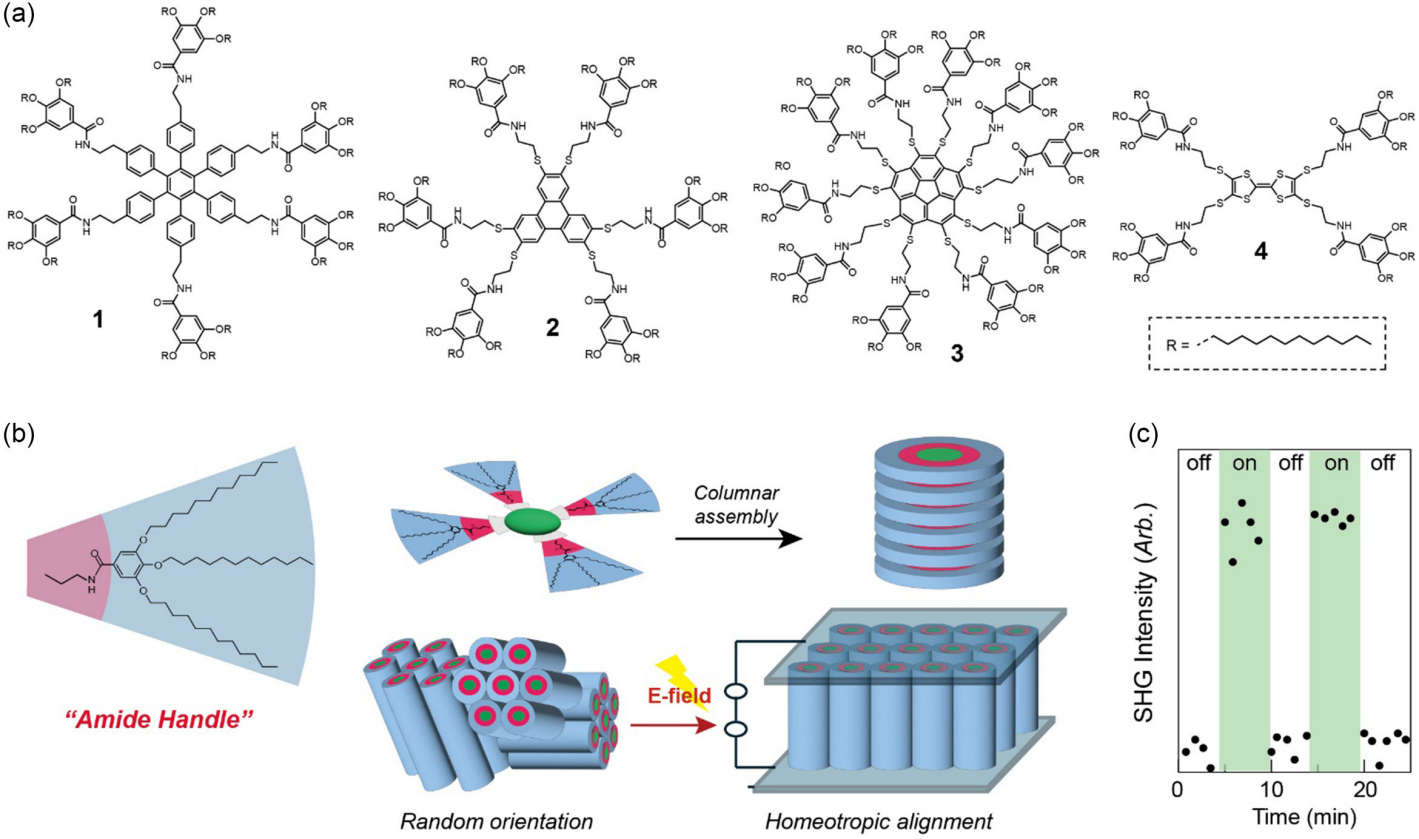
(a) Chemical structures of representative amide‐functionalized discotic mesogens (**1**–**4**; R denotes an alkyl chain). (b) Concept of the “amide handle”: directional amide dipoles and H‐bonding provide an E‐field‐responsive polar motif that promotes columnar self‐assembly and enables electric‐field‐assisted reorientation from randomly oriented polydomains to homeotropic alignment in a sandwich cell. (c) SHG response as a function of time under repeated E‐field on/off cycles. Reproduced from Ref. [54] with permission. Copyright 2011, John Wiley and Sons.

In the early stage of incorporating amide linkages into CLCs, research primarily focused on E‐field‐induced alignment of columnar phases. For example, the Aida group reported electric‐field‐induced alignment of several discotic LCs, i.e., hexaphenylbenzene (**1**), triphenylene (**2**), corannulene (**3**), and tetrathiafulvalene (**4**), bearing amide‐linked peripheral substituents (Figure 6a) [54, 55]. The E‐field alignment of these CLCs was readily evaluated by polarized optical microscopy (POM). Prior to field application, disordered columnar orientations produced characteristic fan‐like textures. Upon applying an AC E‐field (5–25 Vμm^−1^) at elevated temperatures (105–140°C), the initial columns became aligned perpendicular to the electrode surfaces, leading to a uniform dark texture. These observations indicate that the key structural element responsible for alignment is the combination of the amide group and branched paraffinic side chains, referred to as an “amide handle” (Figure 6b).

For several CLC compounds, the field‐induced polar response within the LC phase was further examined by second‐harmonic generation (SHG) measurements [54]. For example, when an AC E‐field (10 Hz, 25 Vμm^−1^) is applied to a discotic LC (**2**) at 110°C, a non‐zero SHG signal is detected under the field, consistent with an induced macroscopic polarization associated with the amide units. However, the SHG signal disappears immediately after removal of the E‐field, indicating that the induced polarization is not retained in the zero‐field state (Figure 6c).

### 3.2 Fan‐Shaped FCLCs With Amide Linkage and Nitrile Core

**FIGURE 7:**
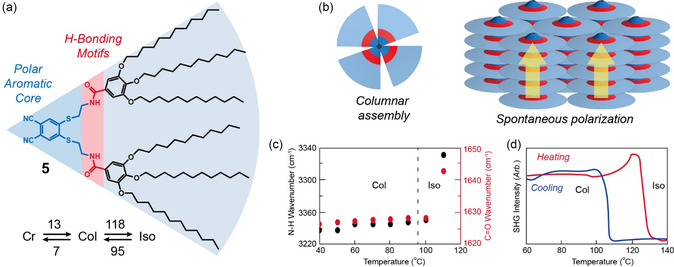
(a) Molecular design of a fan‐shaped mesogen (**5**) combining a polar aromatic core with amide‐based H‐bonding motifs and multiple alkyl chains; the phase sequence is indicated (Cr → Col → Iso). (b) Schematic of columnar self‐assembly and the emergence of spontaneous axial polarization along the column axis. (c) Temperature‐dependent IR signatures (N–H and C = O stretching bands) evidencing the persistence of amide H‐bonding across the columnar phase region. (d) SHG intensity during heating and cooling, showing the appearance of a polar, SHG‐active columnar phase and its thermal reversibility; phase regions are labeled as Cr (crystal), Col (columnar), and Iso (isotropic). Reproduced from Ref. [18] with permission. Copyright 2010, American Chemical Society.

To move beyond alignment control and develop materials capable of generating an axial *P*_s_ along the column axis, the Aida research team designed a fan‐shaped LC (**5**) by combining a strongly polar phthalonitrile core with amide linkers (Figure 7a) [18]. X‐ray diffraction (XRD) revealed that four fan‐shaped molecules assembled into a disc, and these discs further stacked through self‐assembly to form columnar structures (Figure 7b). A distinctive structural feature of this system was that the molecules stacked in an umbrella‐shaped manner, driven by the polar aromatic CN groups in the core together with intermolecular H‐bonding between the amide units (Figure 7c). SHG measurements confirm that the dipoles within the umbrella‐shaped assembly do not cancel each other but instead align cooperatively along the column axis, giving rise to a macroscopic *P*_*s*_ (Figure 7d). However, polarization switching under an applied E‐field was not observed. This is attributed to the rigid intermolecular H‐bond network and the highly congested packing of the polar cores within the column, both of which strongly restrict the rotational and conformational degrees of freedom required for dipole inversion.

**FIGURE 8:**
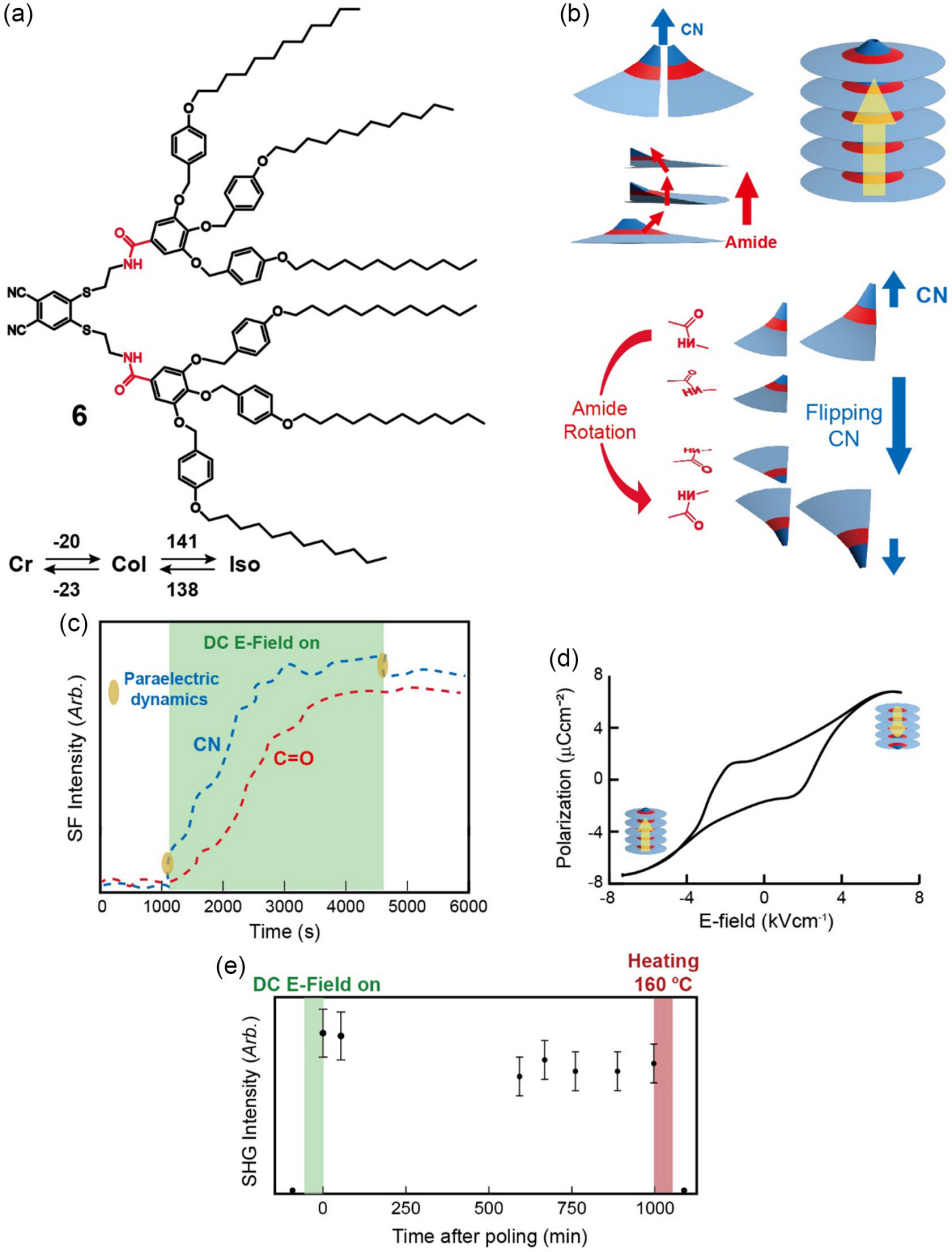
(a) Molecular structure of compound **6** and its phase sequence (Cr→ Col→ Iso; transition temperatures indicated). (b) Proposed mechanism for polarization generation and switching. (c) Time evolution of spectroscopic signatures under a DC electric field (shaded region), showing field‐induced development of polar order (CN and C = O related signals). (d) Representative *P–E* hysteresis loop evidencing reversible polarization switching under an applied field. (e) Temporal evolution of SHG intensity after poling, demonstrating polarization retention; the shaded regions denote periods with DC field applied and the heating event. Reproduced from Ref. [56, 57] with permission. Copyright 2012, The American Association for the Advancement of Science and Copyright 2010, John Wiley and Sons, respectively.

Later, genuine ferroelectric behavior was achieved in an expanded fan‐shaped LC (**6**) in which the peripheral (shell) region of the fan‐shaped architecture was enlarged (Figure 8a) [56]. Despite the increased molecular size, this LC retains the same core–shell organization and the umbrella‐like stacking motif (Figure 8b). Importantly, the increase in steric volume introduced by shell expansion provides sufficient free space to allow rotational motion of the polar cores, and as a consequence, polar switching becomes observable (Figure 8b).

The detailed mechanism of ferroelectric switching in this FCLC system was elucidated by infrared–visible sum‐frequency generation (IV‐SFG) spectroscopy [57]. To selectively probe vibrational modes aligned parallel to the E‐field direction, a “sss” polarization combination (s‐polarized SF, visible, and IR lights) was employed. Analysis of the IV‐SFG spectra shows that, upon application of an E‐field, the signals corresponding to the CN stretching vibration of the phthalonitrile core (2220 cm^−1^) and the C = O stretching vibration of the amide linker (1560 cm^−1^) increase simultaneously, and both reach saturation after approximately 60 min under the applied E‐field (Figure 8c). Notably, the polarization remains after the E‐field is removed, indicating nonvolatile behavior. These results demonstrate that ferroelectric polar switching does not proceed through independent molecular events, but rather through a cooperative process in which core flipping and rotation of the amide H‐bonding network are coupled and occur concurrently (Figure 8b). The fast response (paraelectric‐like behavior) was interpreted as a rapid rise and disappearance of the CN‐related signal upon field application and removal (Figure 8c). This behavior was attributed to a small change in the tilt angle of the core (P_CN_), rather than to full polarization reversal.

The *P–E* hysteresis loop of this FCLC was recorded at 120°C using a triangular waveform at 0.008 Hz. The measurements yield an axial *P*_s_ of 5.8 μCcm^−2^, a *P*_*r*_ of 1.7 μCcm^−2^, and a *E*_c_ of 0.23 Vμm^−1^ (2.3 kVcm^−1^) (Figure 8d). Regarding polarization retention—an essential hallmark of ferroelectricity—SHG experiments confirm that the macroscopic polarization remains stable for approximately 16 h (Figure 8e). On this basis, the authors reported this material as the first FCLC that exhibited both switchable macroscopic polarization and measurable retention. Overall, this FCLC operates only at elevated temperature (120°C) and exhibits relatively slow switching kinetics, yet it functions as an unusual organic ferroelectric that can be driven with an exceptionally low *E*_c_.

### 3.3 Discotic FCLCs With Amide Linkage and Benzotrithiophene Core

**FIGURE 9:**
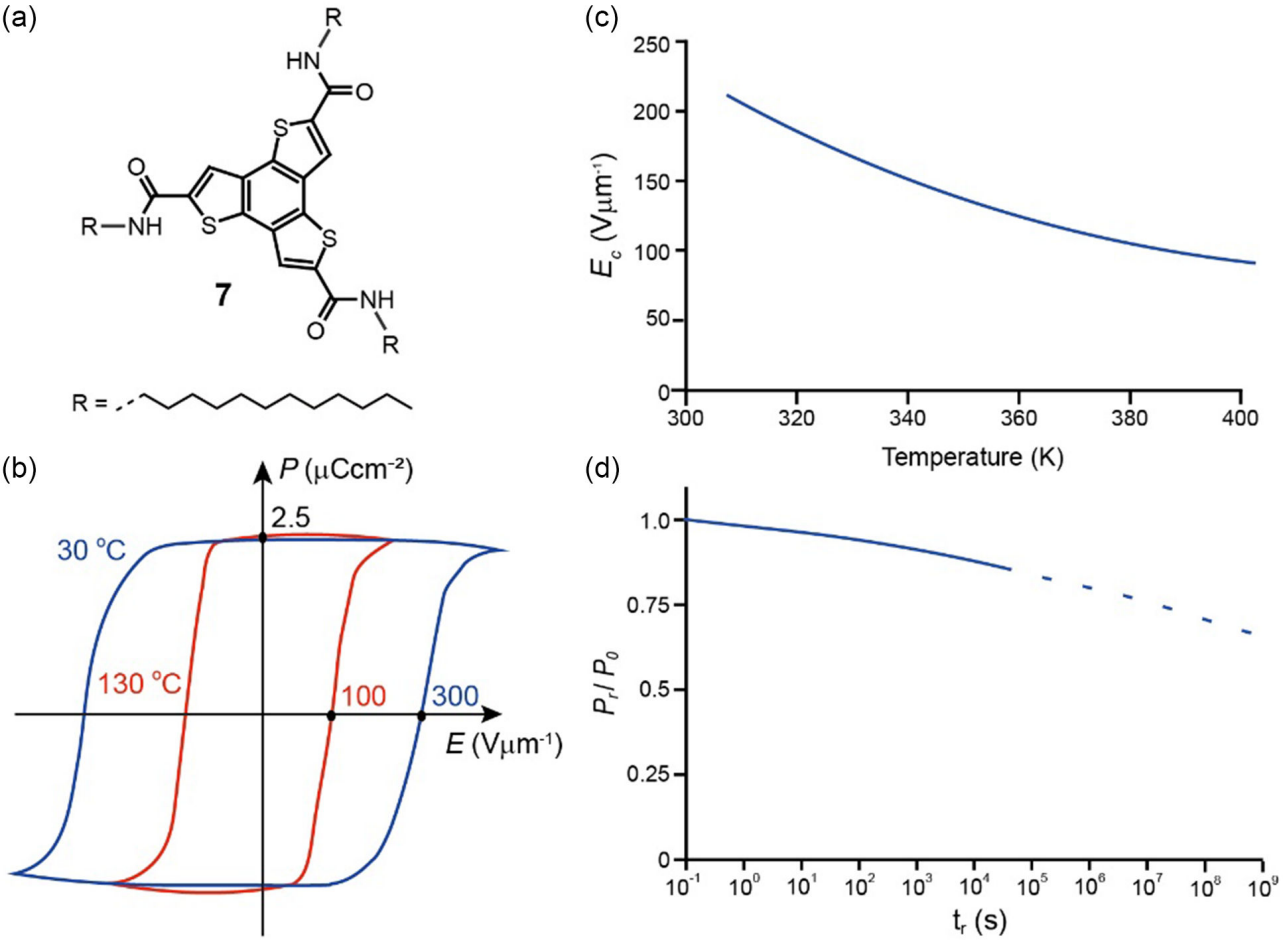
(a) Chemical structure of the benzotrithiophene‐tricarboxamide mesogen (**7**, R = decyl chain). (b) *P–E* hysteresis loops measured at 30°C and 130°C. (c) Temperature dependence of the *E*_c_. (d) Polarization retention behavior expressed as *P*_*r*_/*P*_0_ versus retention time t_r_, indicating long‐lived remnant polarization over extended timescales. Reproduced from Ref. [58] with permission. Copyright 2019, Royal Society of Chemistry.

While many amide‐rich polar CLCs have improved ferroelectric performance primarily through terminal‐chain engineering, an alternative approach has also been explored in which the molecular core is redesigned to enhance the structural order of the ferroelectric assembly. For example, Casellas et al*.* reported that a *C*_3_‐symmetric discotic LC (**7**) based on an extended benzotrithiophene (BTT) core formed highly ordered Col_rec_ and Col_hex_ phases (Figure 9a) [58]. *P–E* hysteresis loops measured from room temperature (RT) up to 130°C reveal a *P*_*r*_ of 2.5 µCcm^−2^, in good agreement with the value expected from geometric considerations (Figure 9b). The *E*_c_ is relatively high—approximately 300 Vμm^−1^ at RT—but decreases to ∼100 Vμm^−1^ at 130°C (Figure 9c). The most notable advance of the BTT‐based FCLC is its markedly improved polarization retention (Figure 9d).

This performance gain could be attributed to the increased structural order brought about by core expansion. Temperature‐dependent WAXS analyses show that the inter‐disc distance remains within a remarkably stable range, changing only from 3.5 Å at low temperature to 4.1 Å at high temperature. The extended BTT core also promoted the formation of larger self‐assembled domains, thereby reducing the density of structural defects. As a result, this FCLC achieves improved polarization retention without requiring an excessive increase in the *E*_*c*_, demonstrating that core engineering can serve as a powerful and complementary route to stabilize ferroelectric order in polar CLCs.

## 4 Benzene‐1,3,5‐Tricarboxamide LCs

**FIGURE 10:**
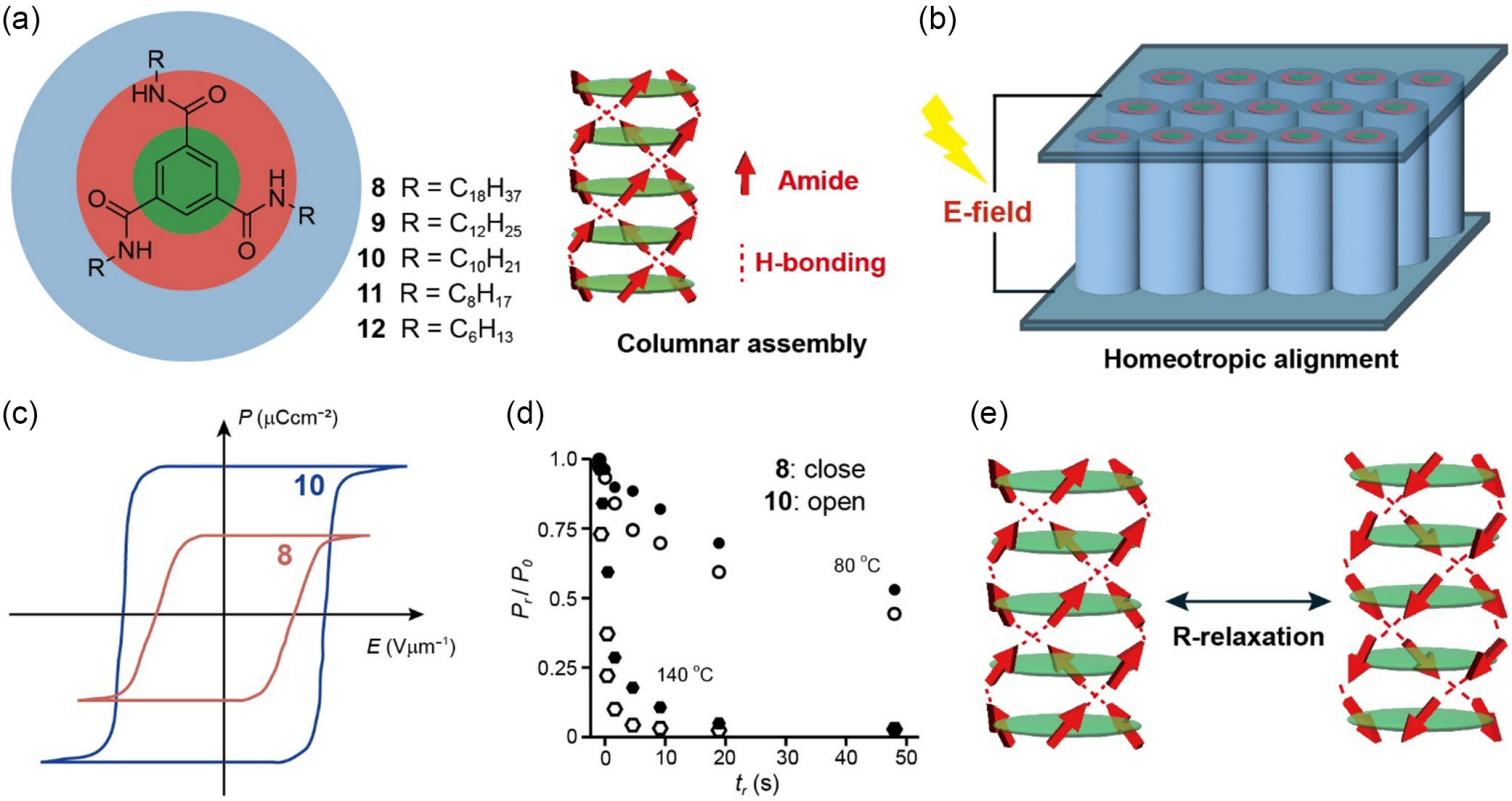
(a) Molecular design of BTA derivatives **8**–**12** with varied linear alkyl chain lengths; cooperative amide H‐bonding promotes polar columnar self‐assembly. (b) E‐field‐assisted processing to achieve homeotropic alignment in a sandwich cell. (c) Representative *P–E* hysteresis loops of **8** and **10**, illustrating ferroelectric switching behavior and remnant polarization. (d) Polarization retention (*P*_*r*_/*P*_0_) as a function of retention time (t_r_) at different temperatures. (e) Schematic of the proposed depolarization pathway (R‐relaxation), involving collective rotational reorganization of amide dipoles along the H‐bonded column. Reproduced from Ref. [63] with permission. Copyright 2012, American Chemical Society.

BTA‐based *C*_3_‐symmetric LCs feature a central benzene core decorated with three radially arranged amide groups [59, 60, 61, 62]. Strong N–H···O=C hydrogen bonding promotes head‐to‐tail 1D stacking of the molecules, leading to robust columnar assemblies (Figure 10a). Within these columns, polar amide bonds can readily align along the column axis, facilitating the formation of a macrodipole along the columnar axis. In addition, the dense 2D packing of columns can stabilize the polarized state and thereby enhance polarization retention. Consequently, BTA derivatives have emerged as a key molecular platform for designing FCLCs in which the polarization can be reversibly switched under an external E‐field. Here, we summarize BTA‐based FCLC studies, focusing primarily on the pioneering contributions of the Kemerink and Sijbesma group.

### 4.1 BTA LCs With Linear Chains

Early BTA‐based CLCs were typically constructed with three linear alkyl chains (Figure 10a). Columns formed by BTA derivatives bearing octadecyl (**8**) and decyl (**10**) chains could be homeotropically aligned in a high‐temperature LC phase (above ∼ 100°C) by applying a DC field of ∼30 Vμm^−1^ (Figure 10b) [63, 64]. For these aligned samples, driving with a triangular waveform (0.1–5 Hz, 400 V_PP_) produces ferroelectric behavior, yielding *P–E* hysteresis loops with a *P*_s_ of 1.3–1.8 µCcm^−2^ and an *E*_c_ of 20–30 Vμm^−1^ (Figure 10c). However, when the bias is removed to evaluate polarization retention, the polarized state relaxes rapidly, and the polarization decays within 2–10^2^ s, highlighting a critical limitation for practical ferroelectric operation (Figure 10d). Importantly, the activation energy extracted from polarization‐retention experiments matches that of the slow relaxation process (R‐relaxation) observed by dielectric relaxation spectroscopy (DRS), indicating that depolarization originates from the collective inversion of the amide dipoles (Figure 10e) [65].

**FIGURE 11:**
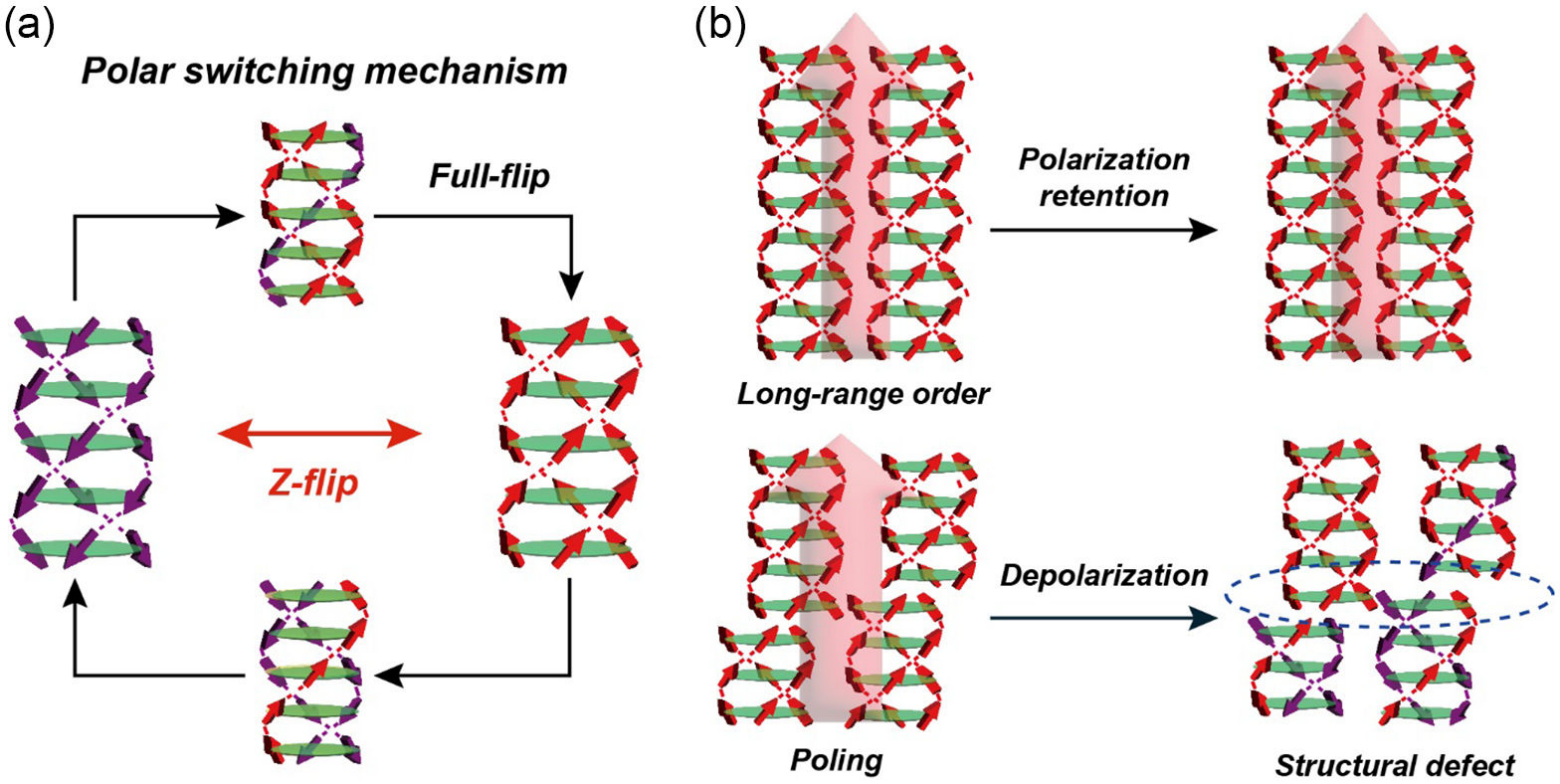
(a) Schematic switching pathways, comparing an energetically favorable Z‐flip process (sequential, domino‐like reorientation along the H‐bonded helical network) with a collective full‐flip mechanism. (b) Conceptual picture of polarization retention in highly ordered assemblies after poling, contrasted with depolarization by structural defects and local disorder that disrupt long‐range cooperativity and promote relaxation of the macrodipole alignment.

To clarify why macroscopic depolarization occurs and how polarization switching proceeds, the Kemerink group performed kinetic Monte–Carlo (kMC) simulations that explicitly considered electrostatic interactions [66]. The simulations reveal that a Z‐flip mechanism, in which molecules switch sequentially along the helical H‐bonded network in a domino‐like fashion, is energetically favored and represents the operative switching pathway (Figure 11a). In addition, polarization relaxation is attributed to intrinsic disorder and structural defects [67]. These led to the design principle that depolarization could be suppressed by reducing internal disorder and strengthening intermolecular coupling to increase the barrier for R‐relaxation (Figure 11b).

**FIGURE 12:**
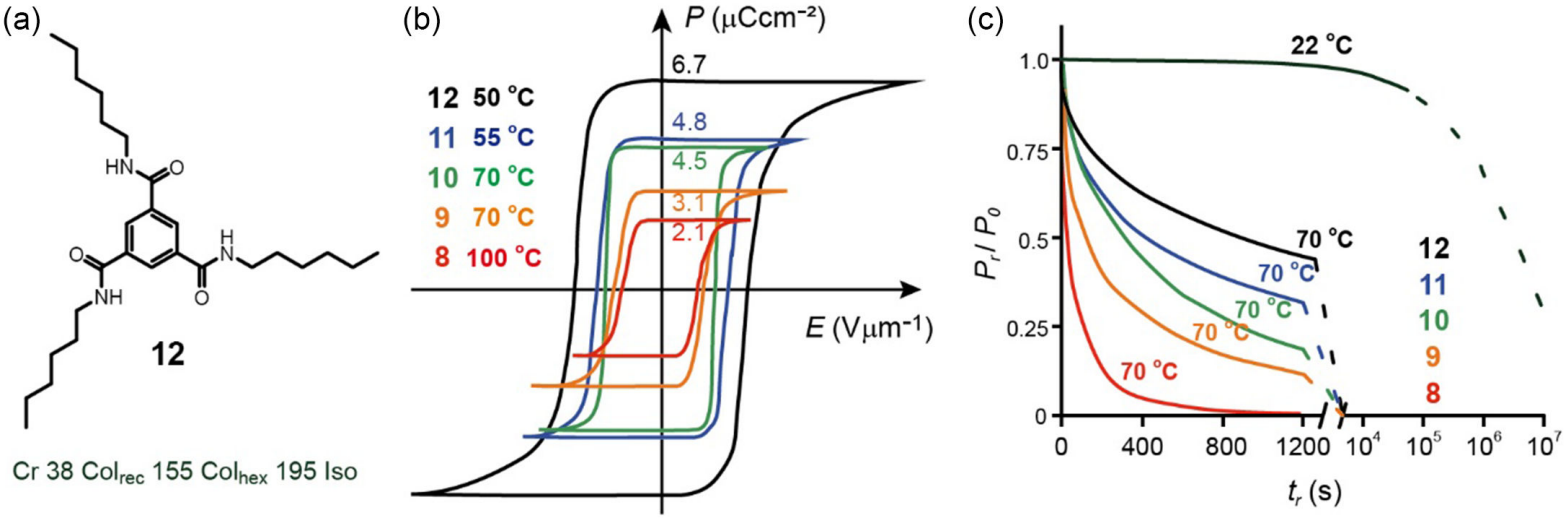
(a) Molecular structure of the shortest‐chain BTA (**12**) and its phase sequence with transition temperatures (Cr → Col_rec_→ Col_hex_→ Iso). (b) *P–E* hysteresis loops for BTA derivatives **8**–**12**, highlighting the increase in remnant polarization and changes in switching behavior as the alkyl chain length decreases. (c) Polarization retention profiles (*P*_*r*_/*P*_0_ vs. retention time t_r_) measured at elevated temperature (70°C) and extrapolated to 22°C. Reproduced from Ref. [68] with permission. Copyright 2017, John Wiley and Sons.

As one molecular strategy to suppress depolarization, the research team shortened the linear alkyl chains down to hexyl chain (Figure 12a) [68]. The *P*_r_ extracted from the *P–E* hysteresis loops increases as the alkyl chains become shorter; notably, **12** exhibits a *P*_r_ of 6.7 µCcm^−2^ (Figure 12b). Importantly, the value for **12** exceeds the value expected from a simple geometric dipole‐density estimate, indicating a cooperative enhancement arising from interactions between macrodipoles. At 70°C, **12** shows an *E*_c_ of 42 Vμm^−1^, and the *E*_*c*_ generally increases with decreasing chain length (Figure 12b). Despite depolarization at 70°C, **12** maintains its polarization for 7 × 10^6^ s (∼80 days) at RT (Figure 12c). This long‐lived retention can be rationalized by the formation of a soft‐crystalline state at RT, where reduced molecular mobility hinders collective dipole reversal. Although these results demonstrate that chain length engineering can enable BTA‐based organic ferroelectrics, BTA derivatives bearing linear chains remain susceptible to thermal fluctuations at elevated temperatures, resulting in rapid polarization loss and motivating further structural optimization.

### 4.2 BTA FCLCs With Branched Chains

**FIGURE 13:**
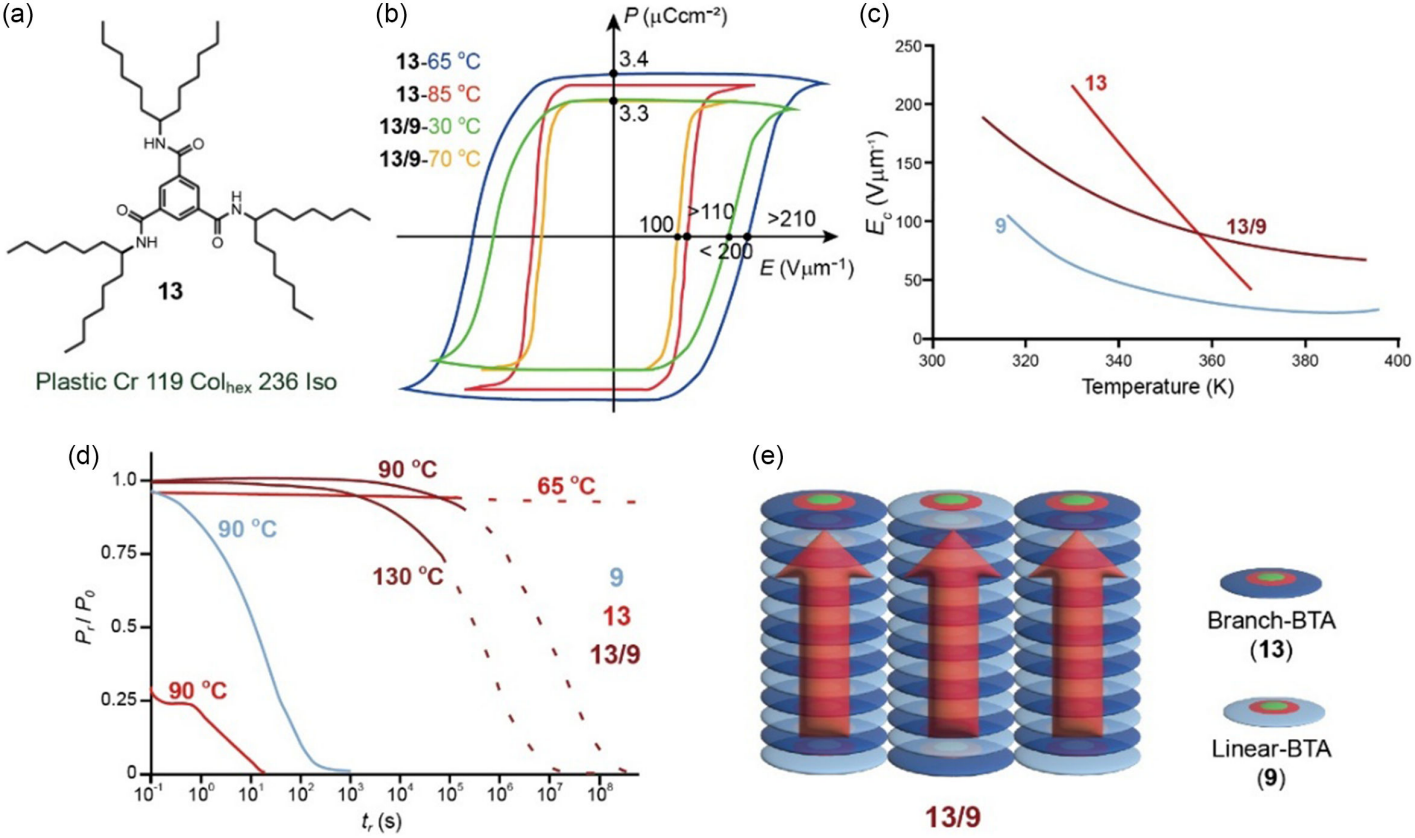
(a) Molecular structure of the branched BTA derivative (**13**) and its phase sequence (plastic Cr→ Col_hex_→ Iso; transition temperatures indicated). (b) *P–E* hysteresis loops of **13** and the **13/9** (1:1) blend measured at different temperatures. (c) Temperature dependence of the *E*_*c*_ for **9**, **13**, and **13/9**, showing that blending reduces the operating field relative to the branched system while maintaining switchability. (d) Polarization retention behavior (*P*_*r*_/*P*_0_ vs retention time t_r_) for **9**, **13**, and **13/9** at elevated temperatures. (e) Schematic illustration of the mixed‐column concept in which branched (**13**) and linear (**9**) mesogens co‐assemble to form columnar structures that combine enhanced long‐range order/defect suppression with a reduced switching barrier. Reproduced from Ref. [69] with permission. Copyright 2019, Royal Society of Chemistry.

To suppress polarization relaxation at elevated temperatures, the Kemerink group proposed a new molecular design (**13**) in which rotation of the amide groups is strongly hindered by introducing a branched 1‐hexylheptyl chain (Figure 13a) [69]. Compared with the linear analogs, the Bragg peaks in its XRD data became sharper and more numerous, indicating a pronounced improvement in long‐range order. This enhanced structural order is attributed to stronger cooperativity in the branched molecules, which form a more ordered plastic‐crystalline phase.

Ferroelectric measurements further support this interpretation [69]. From the *P–E* hysteresis loops, the *P*_r_ of **13** is 3.4 µCcm^−2^, and the *E*_c_ at 65°C reaches 210 Vμm^−1^ (Figure 13b). In contrast to linear BTA derivatives, the substantially increased *E*_c_ for the branched system is rationalized by enhanced steric constraints between columns together with reduced structural disorder, both of which impedes collective dipole reversal (Figure 13c). Polarization‐retention tests demonstrate a dramatic increase in stability: for **13**, no measurable depolarization is observed within the experimental time window at temperatures ≤65°C (Figure 13d). Notably, the improved retention can be explained by superior self‐assembly, as branched BTA forms larger, less defective domains, implying that structural disorder governs the depolarization rate even more strongly than the magnitude of the applied *E*_c_. Although branched BTA thus offered outstanding retention and thermal robustness, it suffered from the practical drawback of requiring high driving voltages (i.e., large *E*_c_) for switching.

To address this trade‐off between polarization retention and operating voltage, the research team implemented a mixed sample (**13/9**) by blending linear and branched BTAs in a 1:1 ratio (Figure 13e) [69]. Specifically, **9** and **13**, which possess similar packing parameters, were combined. The mixture forms a stable Col_hex_ structure without phase separation, and its isotropization temperature increases to ∼250°C, exceeding that of either parent compound. The *P–E* hysteresis loop of the **13/9** blend yields *P*_*r*_ = 3.3 µCcm^−2^ (Figure 13b). The *E*_c_ is reduced to ∼100 Vμm^−1^ at 70°C, while remaining ∼200 Vμm^−1^ at RT (Figure 13c). The blend preserves axial polarization for several months below 90°C, and even at 130°C it maintains polarization for approximately 10 days, demonstrating a balanced improvement in both operational stability and device‐relevant switching conditions (Figure 13d).

### 4.3 Benzene 1,3,5‐Tricarbothioamide LCs

In addition to alkyl‐chain engineering, an alternative strategy has focused on modifying the amide group itself, which serves as the primary origin of ferroelectricity in BTA‐based systems. In this approach, the oxygen atom of the amide was replaced by sulfur to introduce a thioamide unit into the LC mesogen, and the resulting ferroelectric properties were investigated [70]. This structural change increases the dipole moment from 4.0 to 5.1 Debye [71], thereby enhancing the theoretically attainable polarization density per unit volume.

**FIGURE 14:**
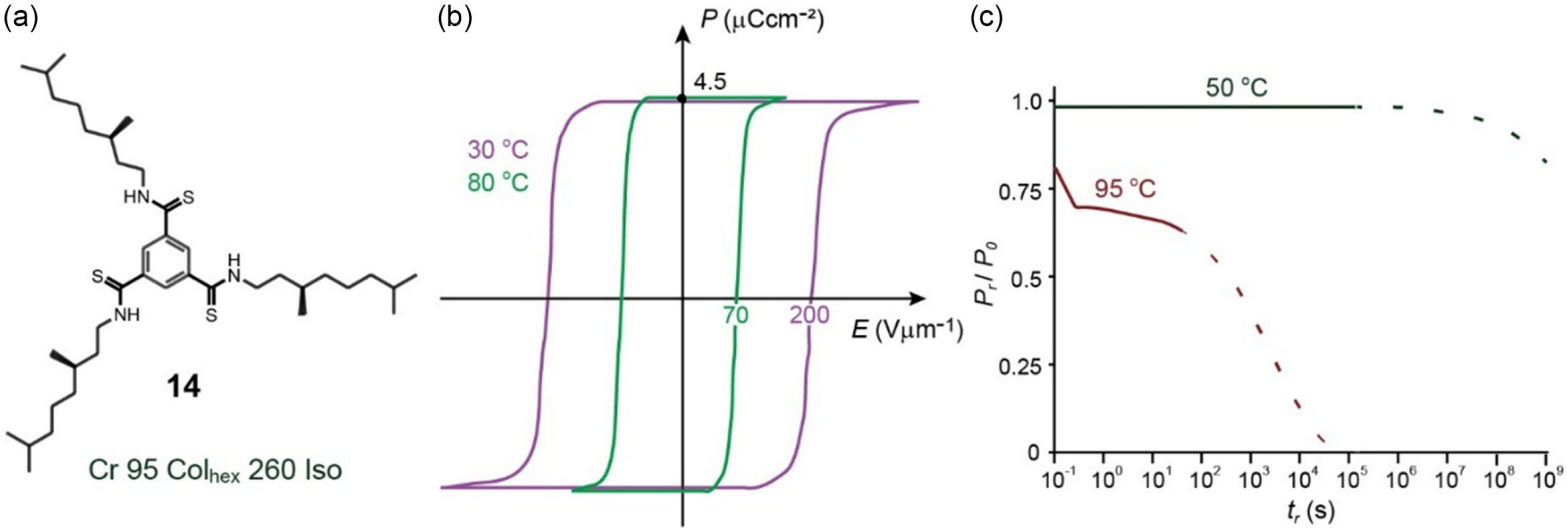
(a) Molecular structure of the thioamide derivative (**14**) and its phase sequence (Cr → Col_hex_→ Iso; transition temperatures indicated). (b) *P–E* hysteresis loops measured at 30°C and 80°C, showing ferroelectric switching with a substantially reduced *E*_*c*_ compared with the amide analog while maintaining a high polarization amplitude. (c) Polarization retention behavior (*P*_*r*_/*P*_0_ vs retention time t_r_) at 50°C and 95°C. Reproduced from Ref. [70] with permission. Copyright 2023, Royal Society of Chemistry.

The branched thioamide derivative (**14**) exhibits a crystalline phase from RT to 95°C, followed by an LC phase from 95 to 260°C, while maintaining hexagonally packed columnar structures in both regimes (Figure 14a). *P–E* hysteresis loops measured over a range of temperatures show that the increased thioamide dipole leads to a high remnant polarization, with *P*_r_ = 4.5 µCcm^−2^ for **14** (Figure 14b). Importantly, at 80°C the *E*_*c*_ of **14** is only ∼70 Vμm^−1^, representing a ∼ threefold reduction in operating voltage compared with **13** (∼210 Vμm^−1^). This decrease is attributed to weaker cooperativity of the inter‐columnar interactions in the solid‐like state for thioamide‐based assemblies relative to the amide analog, enabling polarization switching at a lower energetic cost. Polarization‐retention analyses further indicate that **14** can preserve data for more than 10 years (extrapolated) even at 50°C (Figure 14c). Overall, **14** overcomes the major drawback of branched BTA—namely their excessively high driving voltage (i.e., large coercive fields)—while simultaneously achieving high polarization and polarization retention, making it one of the most balanced ferroelectric performers among BTA‐derived columnar systems.

## 5 Urea Core for Design of Polar CLCs

**FIGURE 15:**
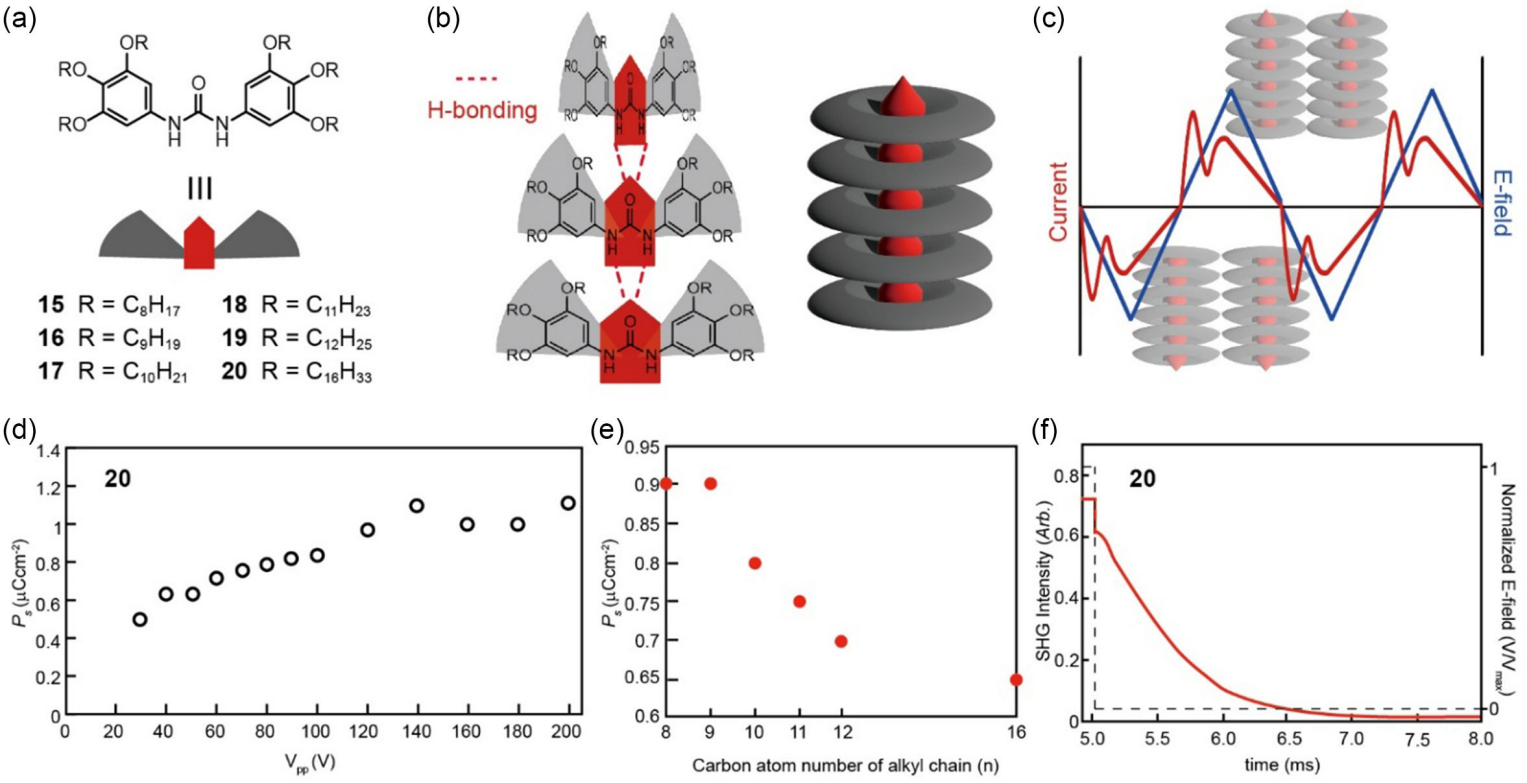
(a) Molecular structures of urea‐based LCs **15**–**20** (R = alkyl chain). (b) Schematic of intracolumnar self‐assembly driven by directional urea H‐bonding, producing a polar stacking motif and an axial macrodipole within the column. (c) Switching‐current response under a triangular‐wave E‐field, consistent with reversible inversion of axial polarization. (d) Dependence of the measured *P*_*s*_ on the applied field amplitude (V_pp_). (e) Alkyl‐chain‐length dependence of *P*_s_. (f) Time‐resolved SHG response under an applied E‐field (normalized field overlaid). Reproduced from Ref. [73, 74, 75] with permission. Copyright 2005, American Chemical Society, Copyright 2010, Taylor & Francis and Copyright 2007, American Physical Society, respectively.

Urea is a functional group that can be regarded as the diamide of carbonic acid. Owing to its high dipole moment (typically >4.5 Debye) [72] and strong H‐bonding ability, urea has been employed as an attractive polar motif for FCLC molecular design (Figure 15a). In urea‐based polar CLC systems, urea units were positioned in the core region to establish a H‐bond‐driven design strategy for generating polar order along the column axis (Figure 15b). Here, we summarize urea core‐based FCLC examples, focusing primarily on the pioneering contributions of the Kishikawa group.

### 5.1 Ferroelectrically Switchable Hexacatenar LC Based on Urea Core

In a representative example developed by the Kishikawa group, urea‐core‐based hexacatenar LCs (**15**‐**20**) with linear chains (Figure 15a) exhibited pronounced homeotropic alignment of the columns under an applied AC E‐field, together with clear polar switching behavior in the mesophase [73]. By applying triangular‐wave E‐fields with varied frequencies (0.1–18 Hz) and field amplitudes (30–200 V_pp_), *P*_s_ values of 1.1–1.57 μCcm^−2^ are obtained, consistent with field‐induced inversion of the axial polarization (Figure 15c).

To further clarify the origin of the observed ferroelectric response, the *P*_s_ of **20** was examined as a function of the applied triangular‐wave amplitude (V_pp_) (Figure 15d). *P*_*s*_ increases with increasing *V*_pp_, and reaches a plateau above ∼120 V_pp_, indicating saturation of axial polarization. In addition, the alkyl‐chain‐length dependence of *P*_*s*_ was systematically investigated (Figure 15e). Increasing the chain length leads to an enlarged column diameter (from 21.9 Å for **15** to 28.8 Å for **20**), accompanied by a decrease in *P*_s_ (from 0.9 μCcm^−2^ for **15** to 0.65 μCcm^−2^ for **20**). This trend supports the interpretation that the measured polarization primarily originates from the urea dipoles, with geometric dilution as the column cross‐section increases. However, despite the observation of switchable polarization under an E‐field, SHG measurements of **20** indicate that the columnar polarization relaxes within milliseconds after E‐field removal [75], revealing that the material behaves effectively as a paraelectric system rather than a retention‐capable ferroelectric (Figure 15f).

### 5.2 Hexacatenar FCLCs Based on Urea Core

**FIGURE 16:**
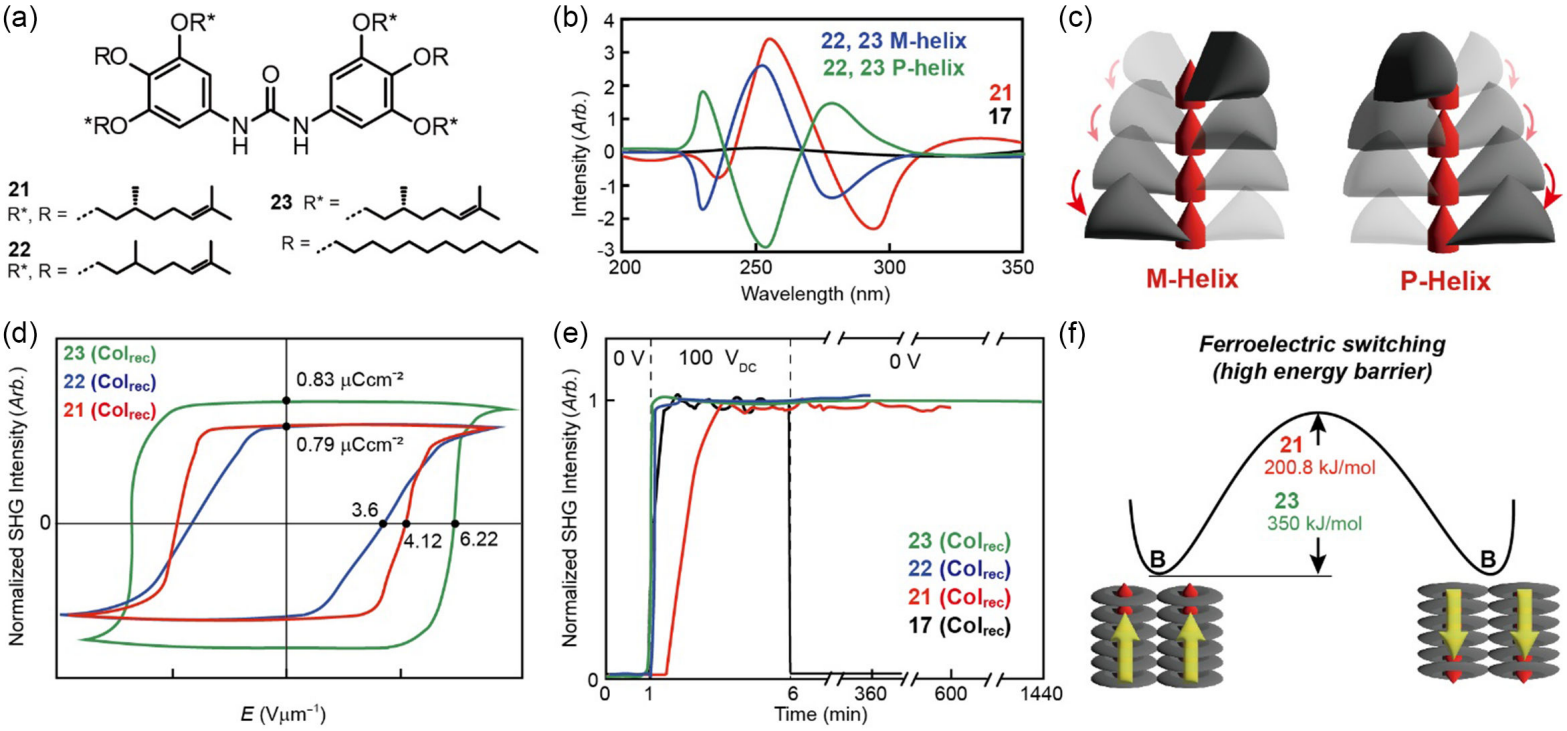
(a) Chemical structures of chiral urea LCs **21**–**23** (R/R^*^ denote alkyl substituents). (b) CD spectra showing opposite helical handedness for the enantiomeric assemblies. (c) Schematic illustration of M‐helix and P‐helix columnar architectures. (d) Field‐dependent SHG hysteresis evidencing polar switching behavior and remnant SHG signals for **21**–**23**. (e) Time evolution of SHG intensity under a DC poling sequence (0 V → 100 V_DC_ → 0 V). (f) Schematic energy landscape for ferroelectric switching, highlighting a high switching barrier associated with helical columnar locking (barriers indicated for **21** and **23**) and the corresponding bistable polarized states. Reproduced from Ref. [76] under the terms of the Creative Commons Attribution Non‐Commercial No Derivatives (CC‐BY‐NC‐ND) license. Reproduced from Ref. [77, 78] with permission. Copyright 2020, John Wiley and Sons and Copyright 2024, American Physical Society, respectively.

To realize macroscopic polarization retention, the Kishikawa team sought to induce a helical columnar architecture. To this end, they synthesized a hexacatenar compound (**21**) in which the terminal achiral chains were replaced with chiral chains (Figure 16a) [77]. XRD and circular dichroism (CD) analyses confirm the formation of strongly twisted M‐helical columns (Figure 16b, c), stabilized by intermolecular H‐bonding. In the helical Col_rec_ phase, the LC exhibits a *P*_*s*_ of 0.79 μCcm^−2^ and an *E*_*c*_ of 4.12 Vμm^−1^ (Figure 16d). Importantly, time‐dependent SHG measurements in the Col_rec_ phase show that the induced macroscopic polarization is retained for more than 10 h without detectable decay (Figure 16e). To rationalize this retention behavior, the authors calculated the switching energy barrier. Compared with the achiral analog (**17**) (∼41.8 kJ/mol), the helical sample (**21**) displays a markedly higher barrier of 200.8 kJ/mol**,** consistent with strongly hindered dipole relaxation and enhanced polarization stability (Figure 16f).

In addition to the above study, the Kishikawa group examined whether ferroelectricity could be preserved when the chiral terminal chains were replaced with racemic chains (Figure 16a) [76]. CD measurements indicate that the racemic sample (**22**) exists as a mixture of M‐ and P‐helical columns (Figure 16b, c), reflecting racemic chiral self‐sorting. In the mixed Col_rec_ phase, the *E*_c_ decreases to 3.6 Vμm^−1^ (Figure 16d), while SHG measurements confirm that polarization retention is maintained for more than 6 h (Figure 16e).

More recently, polarization retention was investigated as a function of the positional placement of the chiral chains (Figure 16a) [78]. Among a series of hexacatenar derivatives with different chiral‐chain positions, only the compound (**23**) with chiral chains located at the 3^rd^ and 5^th^ positions exhibits complete polarization retention over 24 h in the Col_rec_ phase (Figure 16e), as evidenced by SHG. In the Col_rec_ phase, this hexacatenar LC displays an *E*_c_ of 6.22 Vμm^−1^ (Figure 16d) and a high switching energy barrier of 350 kJ/mol (Figure 16f). On the basis of DFT calculations and molecular modeling, this behavior can be attributed to the chiral chains at the third and fifth positions, which optimize intermolecular steric repulsion and suppress fluctuations of the benzene rings, thereby promoting the formation of defect‐free helical columns. However, despite these favorable retention characteristics, polarization switching is restricted at temperatures below 100°C.

### 5.3 Polycatenar RT‐FCLCs Based on Urea Core

**FIGURE 17:**
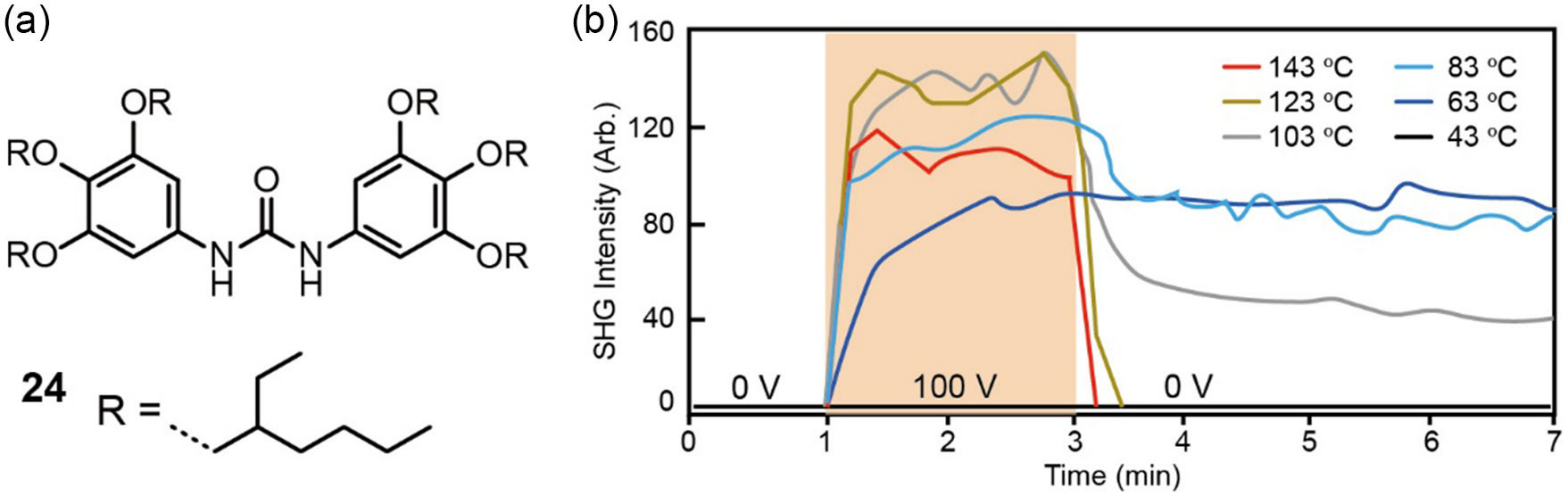
(a) Chemical structure of urea LC (**24**) (R = branched alkyl chain). (b) Time‐resolved SHG intensity during an E‐field on/off protocol (0 V→ 100 V→ 0 V) recorded at different temperatures, showing rapid SHG activation under poling and the temperature‐dependent persistence/decay of the poled polar state after the field is removed. Reproduced from Ref. [79] with permission. Copyright 2020, Oxford University Press.

To lower the temperature range in which polarization switching could occur, a hexacatenar LC (**24**) with an expanded peripheral region was further designed (Figure 17a) [79]. Bulky 2‐ethylhexyl groups were introduced to increase intermolecular spacing and weaken the H‐bonding network. After switching was induced by applying 100 V_DC_ in the resulting Col_hex_ (63–163°C), polarization retention was evaluated by SHG (Figure 17b). The poled state is maintained without detectable decay only at 63°C. Although this represents progress toward lower‐temperature operation, the result is still far from RT‐operation.

**FIGURE 18:**
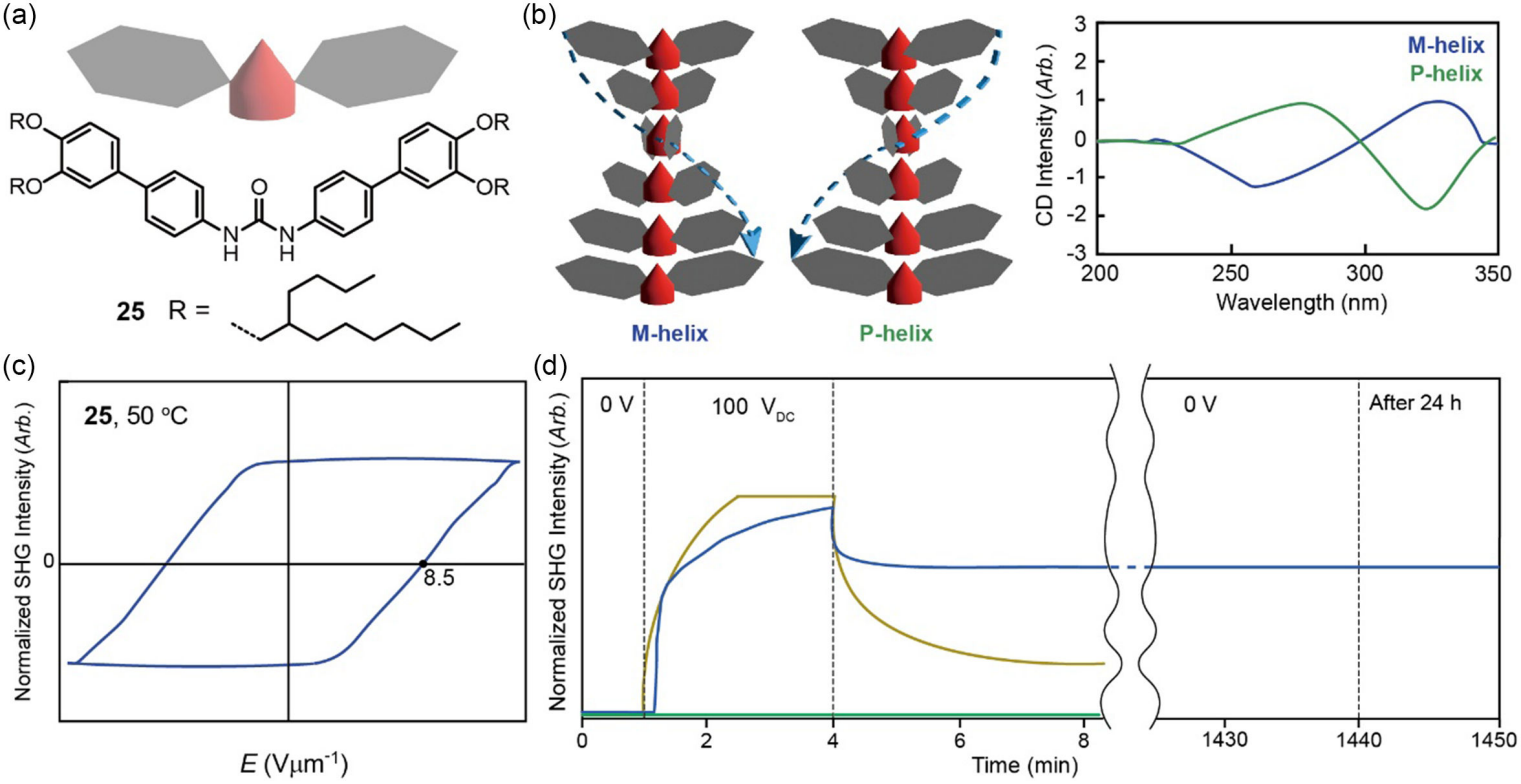
(a) Molecular structure of polycatenar LC (**25**) (R = branched alkyl chain). (b) Schematic M‐ and P‐helical columnar architectures and the corresponding CD response, confirming helical handedness in the self‐assembled columns. (c) SHG hysteresis at 50°C, indicating E‐field‐induced polar switching behavior. (d) Time evolution of SHG intensity during DC poling (0 V → 100 V_DC_ → 0 V) at 50°C. Reproduced from Ref. [80] with permission. Copyright 2023, American Physical Society.

Before fully realizing RT‐ferroelectric operation, the Kishikawa group attempted to mitigate the inherent fluidity of LC materials by introducing a “save‐state” strategy: After polar switching, the sample was cooled to a temperature at which polarization decay could be suppressed, thereby preserving the polarized state [80]. In this approach, the number of branched chains was reduced from six to four, and a new urea LC (**25**) was designed with an elongated molecular framework by incorporating a biphenyl unit and extending the chain length (Figure 18a). Owing to steric twisting of the biphenyl group, the material forms P‐ and M‐helical columns (Figure 18b), as confirmed by CD spectroscopy and 2D XRD. Polarization switching becomes possible at 50°C, with an *E*_c_ of 8.5 Vμm^−1^ (Figure 18c). At the same temperature, SHG measurements indicate that polarization retention is maintained for more than 1 day (Figure 18d). However, switching is not achievable at 30°C. Instead, although operationally cumbersome, the polarization state can be “saved” by cooling the sample after switching.

**FIGURE 19:**
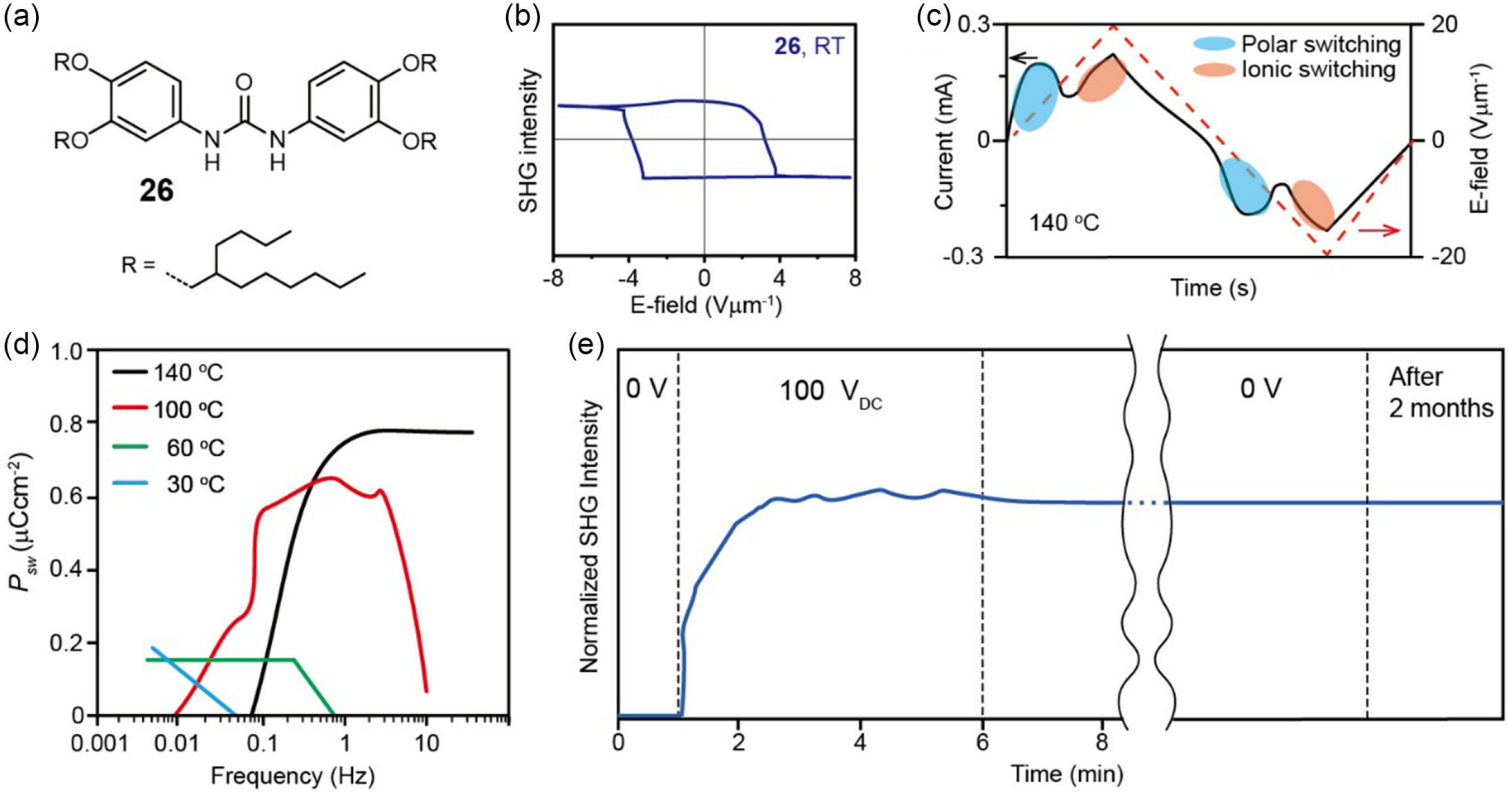
(a) Chemical structure of polycatenar LC (**26**) (R = branched alkyl chain). (b) SHG hysteresis loop of **26** at RT. (c) Schematic illustration of the waveforms of the voltage applied to the sample (dotted red line) and the resultant current profile (solid black line), defining the P_r_. (d) Plots of P_sw_ values of **26** against frequency at various temperatures. (e) Time‐resolved SHG intensity during DC poling (0 V→ 100 V_DC_→ 0 V). Reproduced from Ref. [81] with permission. Copyright 2024, American Physical Society.

To achieve the practical goal of true RT‐operation (RT‐FCLC)**,** the group further refined the molecular design [81]. Specifically, the biphenyl unit was replaced with a simpler phenyl group, while maintaining four branched chains (e.g., 2‐butyloctyl), yielding a polycatenar LC (**26**) (Figure 19a). With this modification, the compounds exhibit a Col_hex_ phase even at RT. Switching measurements using triangular waveform and SHG interferometry reveal an exceptionally low *E*_c_ of ∼ 3.5 Vμm^−1^ at RT (Figure 19b). The switchable polarization (*P*_*sw*_) is ∼0.8 μCcm^−2^ at 140°C, close to the *P*_*s*_ predicted by density functional theory calculations (Figure 19c). In contrast, *P*_sw_ at RT obtained from a 0.01 Hz triangular‐wave measurement is less than 0.2 μCcm^−2^ (Figure 19d). This reduced value is attributed either to slow switching kinetics caused by high viscosity and/or to incomplete macroscopic alignment, which likely leads to an underestimation of the intrinsic polarization. Importantly, SHG measurements performed after RT‐switching confirm long‐term retention of the poled state for ∼2 months (Figure 19e). Overall, these studies demonstrate that rational molecular redesign enables the development of urea‐based ferroelectric materials that are operable at RT with robust polarization retention.

## 6 Triazole Linkage for Design of Polar CLCs

The 1,2,3‐triazole unit is not only readily accessible via copper‐catalyzed azide–alkyne cycloaddition (CuAAC) [82, 83, 84], but also intrinsically highly polar, exhibiting a large dipole moment (4.55 Debye) [85, 86]. Owing to its asymmetric heteroaromatic ring, it can act as both a H‐bond donor (C–H) and acceptor (N), providing directional, programmable intermolecular interactions. In contrast to widely used amide‐based, non‐aromatic polar linkers, 1,2,3‐triazole is a rigid aromatic ring that can serve as a structural element of the mesogenic core itself. Compared with amide‐type functionalities, this aromatic polar group has been incorporated into E‐field‐responsive CLCs only relatively recently. In 2025, it was first employed by the Cho group to develop a genuine FCLC. Here, we highlight recent advances in E‐field‐oriented CLCs and FCLCs based on 1,2,3‐triazole linkages, with a primary focus on the pioneering contributions of the Cho group.

### 6.1 E‐Field‐Induced Orientation of CLCs based on 1,2,3‐Triazole Linkages

**FIGURE 20:**
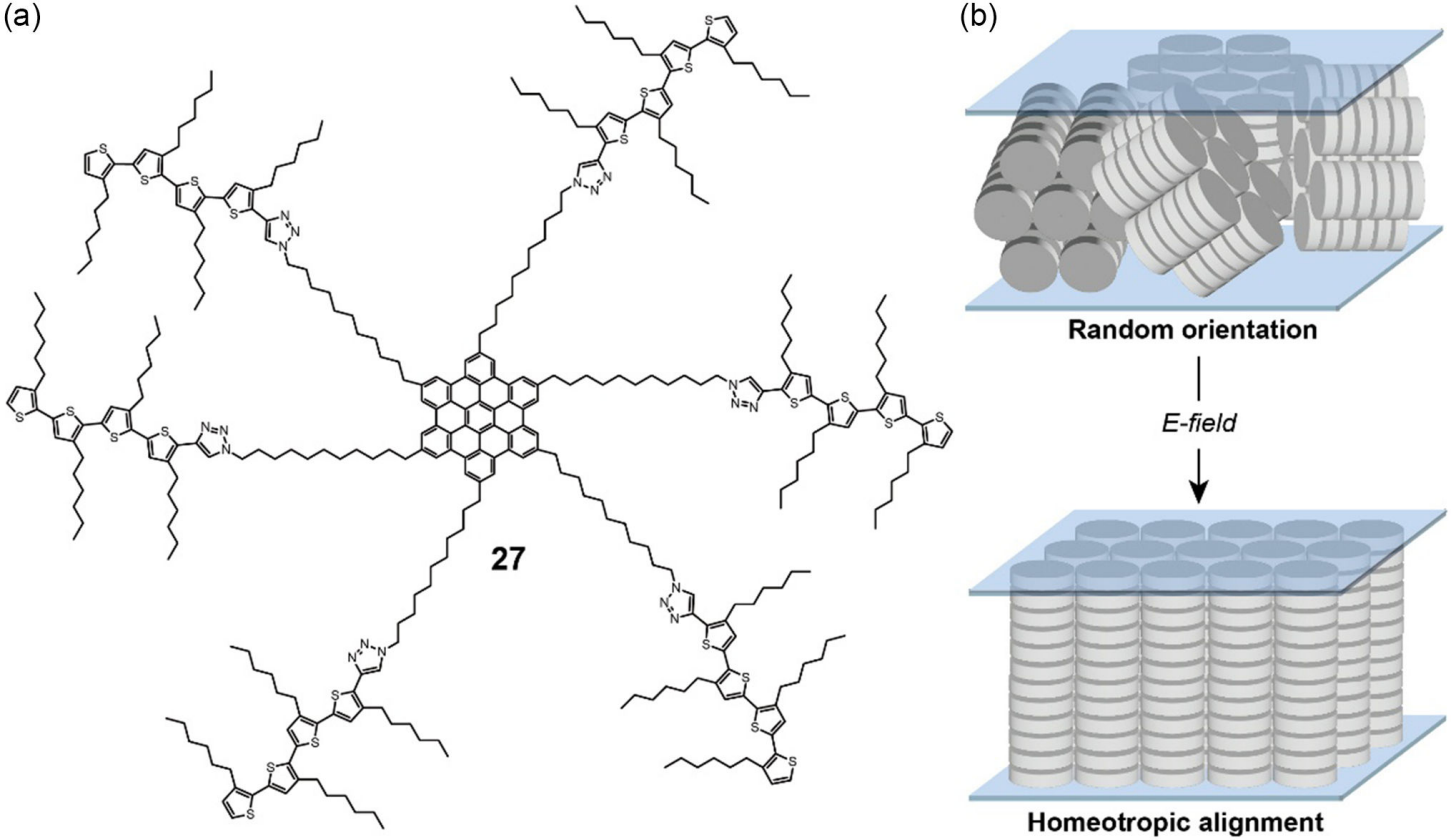
(a) Molecular structure of discotic LC (27), in which a discotic core is functionalized with oligothiophene units through 1,2,3‐triazole linkers. (b) Schematic of E‐field‐assisted orientation in a sandwich cell.

In 2014, Hu et al*.* reported a discotic LC (**27**) by attaching six oligothiophene tails to a hexa‐peri‐hexabenzocoronene (HBC) core through 1,2,3‐triazole linkers via click chemistry and demonstrated a CLC whose orientation can be readily controlled by an electric field (Figure 20) [87]. The compound forms a stable hexagonal columnar (Col_hex_) phase over a wide temperature range, while crystallization is suppressed to below RT, allowing a supercooled Col_hex_ phase to be obtained. In a standard ITO‐coated glass cell, cooling alone produces a non‐uniform planar alignment; however, applying a 2 Hz square‐wave field (±30 Vμm^−1^) at 80°C converts the sample to a high‐quality homeotropic alignment, and the field‐induced orientation persists after the field is removed. Using an in‐plane switching (IPS) cell (10 μm gap), uniform planar alignment is also achieved by applying an in‐plane field (±15 Vμm^−1^) during cooling. The electro‐optic switching was interpreted not as ferroelectric switching but as dielectric switching governed by a positive low‐frequency dielectric anisotropy (Δ*ε*), which was attributed to dipolar reorientation of the triazole–oligothiophene chains.

**FIGURE 21:**
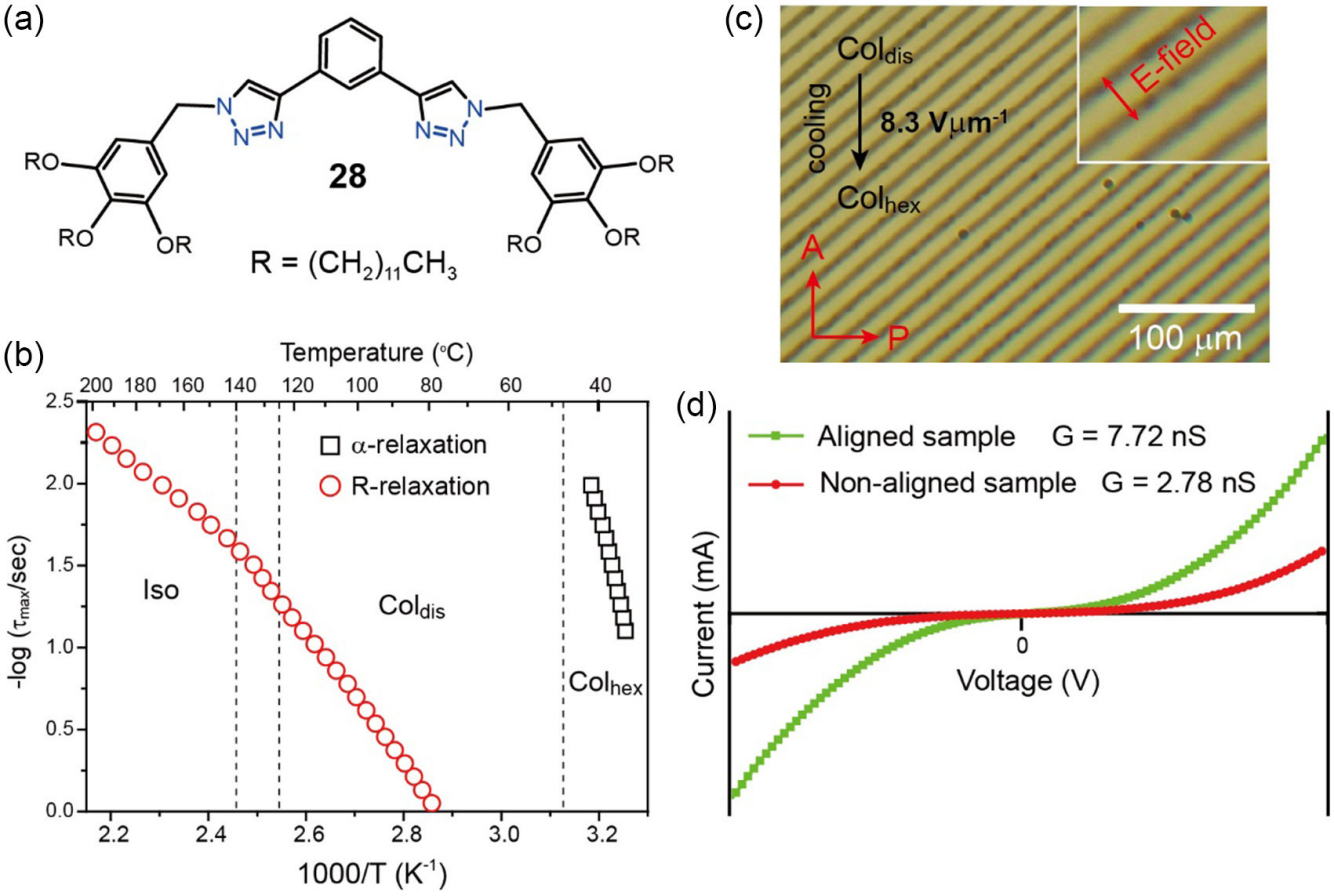
(a) Chemical structure of bent‐core LC (**28**). (b) Arrhenius‐type DRS analysis showing an α‐relaxation in the ordered Col_hex_ phase and an additional R‐relaxation (flipping/reorientation mode) activated in the Col_dis_ and Iso states. (c) POM image of a uniformly aligned texture obtained by a cooling‐alignment method (cooling from Col_dis_ to Col_hex_ under an applied field; ±8.3 Vμm^−1^). (d) Current–voltage characteristics comparing aligned and non‐aligned samples, showing higher conductance for the aligned state (G values indicated), consistent with improved charge‐transport pathways along the oriented columns. Reproduced from Ref. [88] with permission. Copyright 2017, Royal Society of Chemistry.

In 2017, the Cho group reported an E‐field‐responsive bent‐core LC (**28**) in which a 1,2,3‐triazole unit served as a key mesogenic element (Figure 21a) [88]. This LC forms a Col_hex_ near RT and to transform upon heating into a disordered columnar phase (Col_dis_) with only short‐range correlations. DRS analysis reveals that the Col_hex_ phase permits mainly in‐plane rotation of the aromatic core (α‐relaxation), whereas in the Col_dis_ phase, an additional relaxation mode (R‐relaxation; flipping motion) becomes activated (Figure 21b), allowing triazolyl units to reorient along the E‐field direction. Leveraging these dynamics, a “cooling‐assisted alignment method” was proposed, in which the sample was cooled from Col_dis_ to Col_hex_ under an applied field. Compared with isothermal alignment, this method enabled uniform vertical alignment within  ∼1 min under a relatively low field (10 Vμm^−1^) and notably achieved complete vertical alignment even in bulk films as thick as 260 μm, as confirmed by in situ XRD. Moreover, when an in‐plane switching (IPS) cell was used to apply a field parallel to the substrates, the columnar domains could be uniformly aligned in‐plane (Figure 21c), yielding *a* ∼ 2.8‐fold enhancement in electrical conductivity relative to non‐aligned samples (Figure 21d)—highlighting the potential for device‐level performance improvements.

**FIGURE 22:**
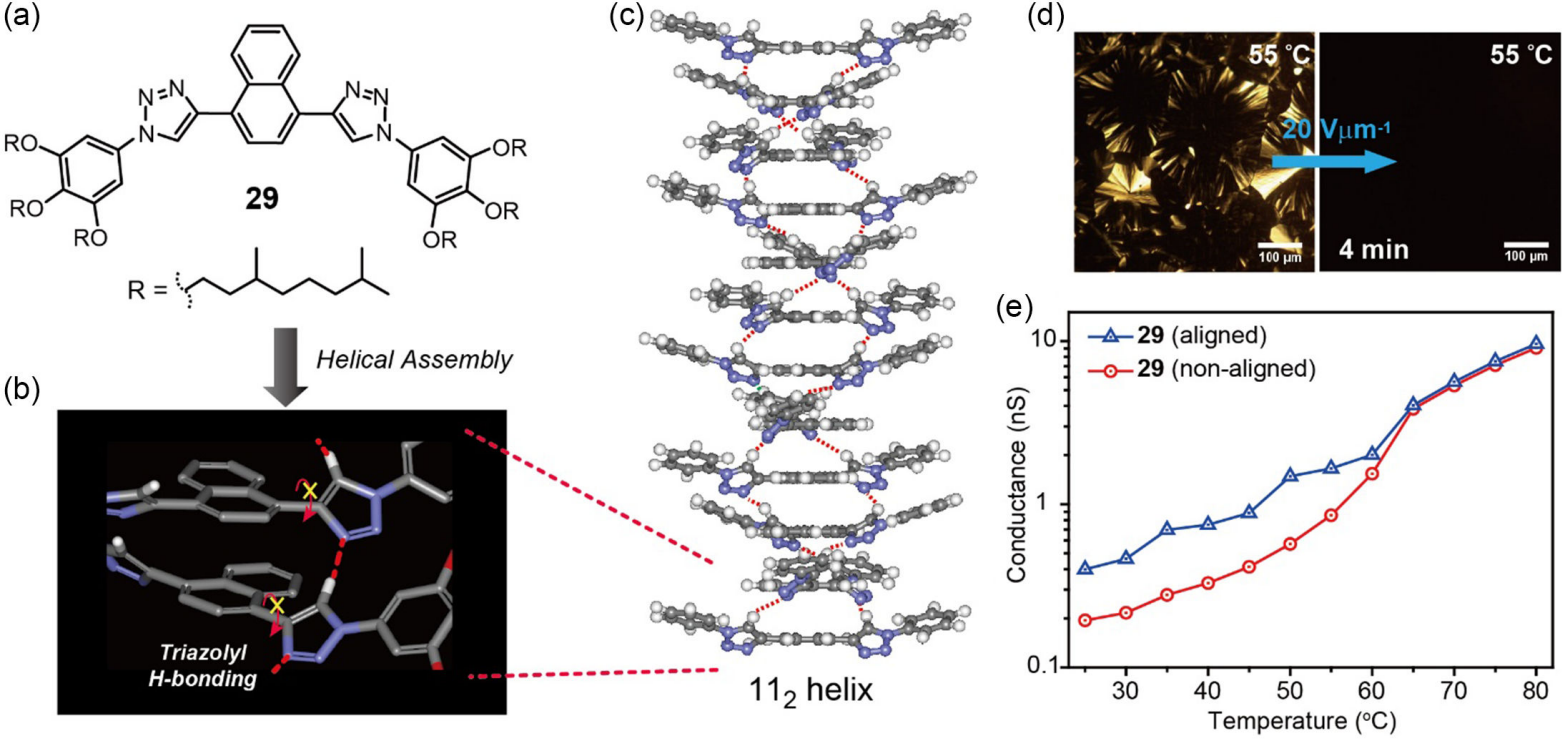
(a) Molecular structure of hexacatenar LC (**29**) (R = branched alkyl chain). (b) Schematic of triazolyl C–H···N H‐bonding that promotes a directional intracolumnar network, enabling helical self‐assembly. (c) Proposed intracolumnar 11_2_ helical stacking model derived from structural/energetic analysis. (d) POM images showing transformation from a polydomain texture to a uniformly aligned dark state upon applying an electric field (20 V μm^−1^) at 55°C for 4 min. (e) Temperature‐dependent conductance of aligned versus non‐aligned samples, demonstrating enhanced charge transport in the aligned columnar state. Reproduced from Ref. [89] with permission. Copyright 2021, American Chemical Society.

The Cho group elucidated the origin of the improved columnar alignment enabled by incorporating 1,2,3‐triazole [89]. In this study, intracolumnar helical order emerged for the triazole‐based hexacatenar LC (**29**) (Figure 22a), driven by N···H–C interactions between neighboring triazolyl groups (Figure 22b). XRD combined with energetic simulations suggested a 11_2_ helix with a helical axis offset from the molecular long axis (Figure 22c). A key structural factor is the tilted conformation enforced by steric repulsion between triazolyl C–H and adjacent aromatic C–H groups; this conformation promotes formation of an H‐bond network while simultaneously enabling rotational flexibility of the linker. Uniform vertical alignment can be achieved within 4 min at 20 Vμm^−1^ (Figure 22d). Importantly, aligned samples of **29** display  ∼2‐fold higher conductivity in the LC regime below 60°C (Figure 22e), demonstrating that simple E‐field alignment can effectively improve one‐dimensional transport pathways along the columns.

### 6.2 Piezoelectric Hexacatenar LCs Based on 1,2,3‐Triazole Linkages

**FIGURE 23:**
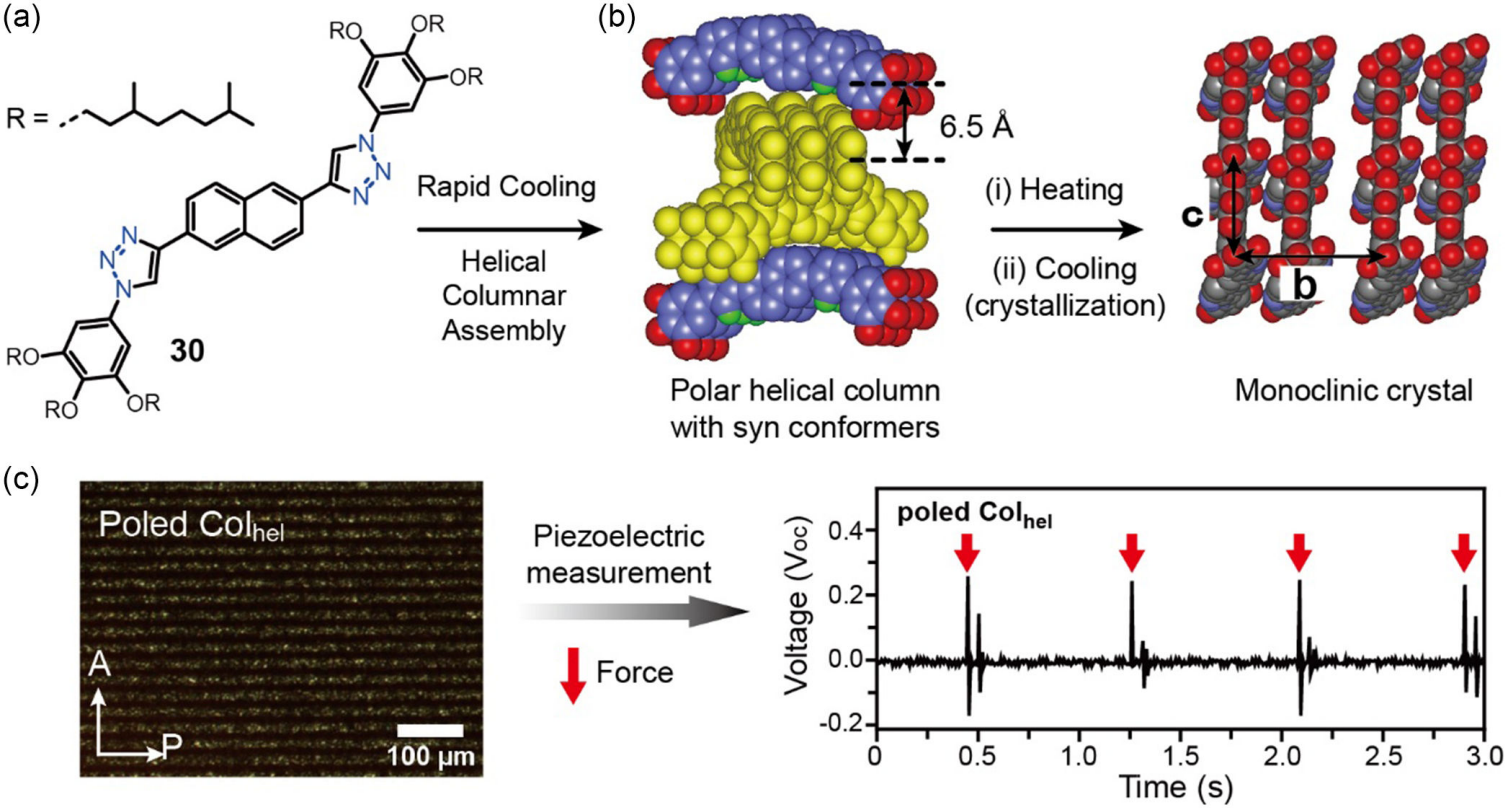
(a) Molecular structure of hexacatenar LC (**30**). (b) Structural models illustrating a polar helical column and the thermodynamically stable monoclinic crystal. (c) POM image of a macroscopically aligned, poled Col_hel_ sample in an IPS cell (left) and the corresponding piezoelectric measurement (right), where an open‐circuit voltage (*V*_oc_) is generated upon periodic mechanical loading (arrows), demonstrating a reproducible piezoelectric response of the poled helical columnar state. Reproduced from Ref. [90] with permission. Copyright 2021, Royal Society of Chemistry.

Beyond alignment control, a triazole‐based helical columnar polymorph was also reported to exhibit piezoelectricity at RT after poling [90]. A naphthalene derivative (**30**) bearing a 1,2,3‐triazolyl linker was shown to form two RT polymorphs: a metastable Col_hel_ and a thermodynamically stable monoclinic crystal (Figure 23a,b). The key finding is that the piezoelectric response depends strongly on whether the Col_hel_ phase is macroscopically aligned. Rapid cooling yields Col_hel_, where a head‐to‐tail triazolyl H‐bond network constrains syn conformers in parallel along the column axis, generating a polar helical order with a long lifetime at RT (Figure 23b). Piezoelectric measurements were performed using an IPS ITO cell: Col_hel_ was poled under a DC E‐field to align columns along the field direction, and the open‐circuit voltage (V_oc_) generated under mechanical stress was monitored. Poled Col_hel_ exhibits reproducible *V*_oc_ peaks (Figure 23c).

### 6.3 Ferroelectrically Switchable Hexacatenar LCs Based on 1,2,3‐Triazole Linkages

**FIGURE 24:**
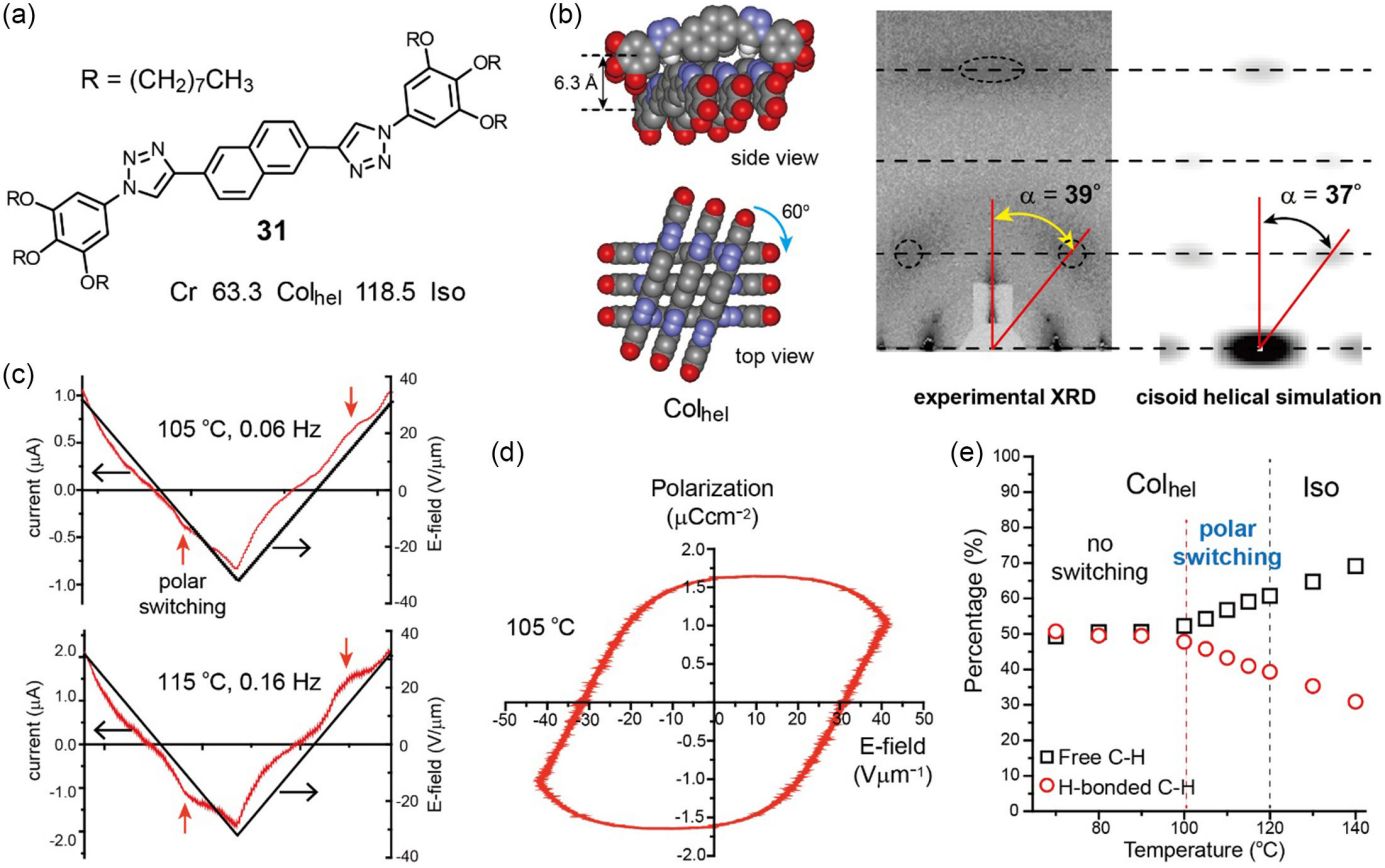
(a) Chemical structure of the naphthalene–triazole mesogen (**31**) (R = octyl group) and its phase sequence (Cr→ Col_hel_→ Iso; transition temperatures indicated). (b) Proposed cisoid helical columnar packing model and comparison between experimental 2D XRD and simulated diffraction patterns. (c) Switching‐current responses under a triangular‐wave AC electric field at 105°C and 115°C, highlighting current peaks associated with polar switching. (d) DWM‐derived *P–E* hysteresis loop at 105°C demonstrating reversible polarization inversion, with *P*_*s*_ and *E*_*c*_ defined in the plot. (e) Temperature‐dependent evolution of the triazolyl C–H populations (free vs H‐bonded). Reproduced from Ref. [91] with permission. Copyright 2019, John Wiley and Sons.

In 2019, the Cho group reported ferroelectric switching in a hexacatenar LC (**31**) featuring a naphthalene core and triazole linkers (Figure 24a) [91]. Owing to an extended H‐bonded network between the triazolyl units, this compound forms a double‐stranded Col_hel_ phase (Figure 24b). X‐ray simulations suggest that the helical assembly is built from cisoid conformers with a non‐zero dipole moment (Figure 24b), producing axial polarization.

Upon application of an AC electric field, pronounced switching‐current peaks are observed only within the Col_hel_ temperature window (105–115°C) under a triangular waveform (Figure 24c). The ferroelectric response was further quantified by the double‐wave method (DWM), yielding well‐defined *P–E* hysteresis loops. At 105°C, the *P–E* loop shows a saturated *P*_*s*_ of 1.62 μCcm^−2^ with a *E*_c_ of 30 Vμm^−1^ (Figure 24d). At 115°C, *P*_*s*_ decreases to 1.37 μCcm^−2^ and *E*_c_ to 20.9 Vμm^−1^, indicating that the switching barrier decreases with increasing temperature. The close agreement between the measured *P*_s_ and the theoretical value supports a rotation‐driven switching mechanism of the triazole‐based polar groups (axial polarization inversion), rather than ionic migration or charge‐injection artifacts.

The temperature dependence of switching was rationalized in terms of changes in H‐bond strength. Temperature‐dependent IR measurements show systematic variations in the triazolyl C–H bands (Figure 24e). The IR results indicate that at lower temperatures, a stronger H‐bond network suppresses rotational motion of the triazole units and thus eliminates switching, whereas rotation‐driven switching becomes activated only in the high‐temperature Col_hel_ regime where H‐bonding is partially weakened. By exploiting the synthetic accessibility of triazole units via click chemistry, this study established a clear structure–dynamics–ferroelectric switching relationship.

### 6.4 RT‐Operable Discotic FCLC Based on 1,2,3‐Triazole Linkages

**FIGURE 25:**
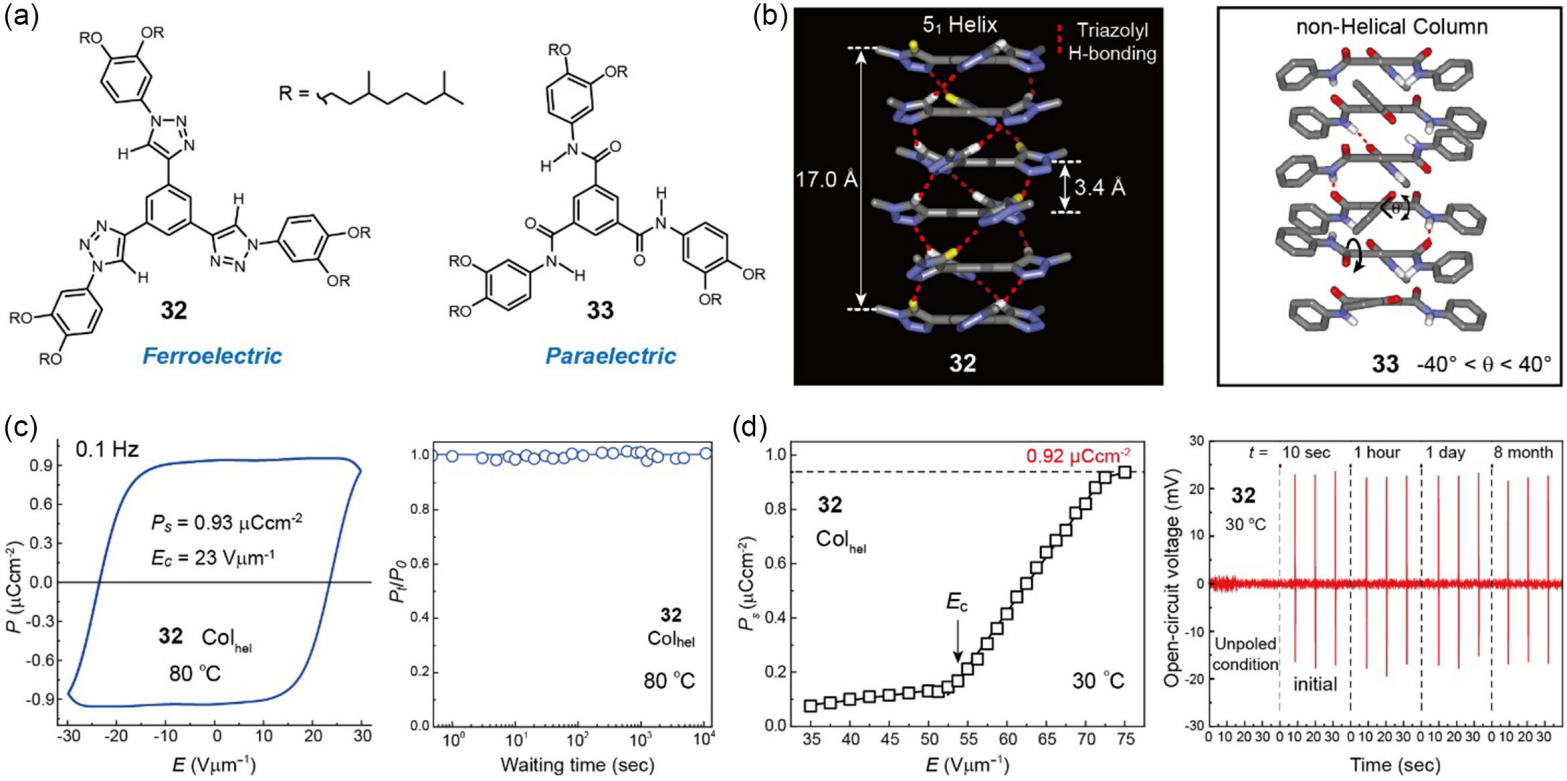
(a) Chemical structures of star‐shaped *C*_3_‐symmetric LCs: triazole‐linked compound **32** (ferroelectric) and the amide‐linked analog **33** (paraelectric). (b) Structural models comparing the 5_**1**_ helical column formed by **32** through triazolyl H‐bonding (left) with the nonhelical columnar packing of **33** (right). (c) DWM‐derived *P–E* hysteresis loop of **32** in the Col_hel_ phase (80°C) and the corresponding polarization‐retention data (*P*_*t*_/*P*_0_) versus waiting time. (d) RT switching characteristics of **32** (30°C): polarization–field response showing ferroelectric switching with *E*_*c*_ and *P*_*s*_ (0.92 μCcm^−2^) indicated, and piezoelectric V_oc_ traces confirming long‐term nonvolatile polarization retention over extended periods (up to months) after poling. Reproduced from Ref. [92] with permission. Copyright 2025, John Wiley and Sons.

For practical memory devices such as FeRAM, FCLC materials must exhibit ferroelectricity at RT, and the polar columnar domains must not undergo partial polarization relaxation after the E‐field is removed [78]. However, even in previously reported FCLC systems, undesirable polarization inversion in a fraction of polar domains upon field removal has been recognized as a persistent limitation. Very recently, the Cho group demonstrated an effective strategy to address this issue by employing a *C_3_*‐symmetric discotic mesogen (**32**) incorporating bulky 1,2,3‐triazole linkers [92]. In this study, they compared two analogous discotic LCs—one with 1,2,3‐triazole (**32**) and the other with an amide linker (**33**) (Figure 25a) and validated the hypothesis that the polar linker itself governed RT ferroelectricity. The bulky triazole linker stabilizes an asymmetric Col_hel_ phase at RT, enabling “genuine” ferroelectric behavior, whereas the amide analog remains nonhelical Col_hex_ (Figure 25b). Even when switching‐like signals are detected in the Col_hex_ of **33**, polarization rapidly decays and the material behaves effectively as paraelectric.

Quantitatively, in the Col_hel_ phase (80°C) of **32**, DWM yields *P*_*s*_ = 0.93 μCcm^−2^ with saturation behavior and *E*_c_ = 23 Vμm^−1^ (Figure 25c), indicating a rotation‐based switching mechanism. In contrast, **33** can switch with a lower *E*_c_ (15 Vμm^−1^) through amide bond rotation, but the nonhelical packing permits greater freedom of polar groups, leading to depolarization. Crucially, **32** shows no polarization decay: In a modified DWM protocol, *P*_*t*_ */P*_0_ remains 1.0 even after 10,800 s, and piezoelectric open‐circuit voltage measurements confirm robust nonvolatile polarization under zero field (Figure 25c). The authors concluded that forming a helical columnar architecture is essential for realizing FCLC behavior, because the asymmetric H‐bond network produces by a 5_1_ helix and a tilted triazole geometry suppresses conformational relaxation and locks the axial macrodipole alignment (Figure 25b).

From an application standpoint, RT operation is the final requirement. For **32**, rectangular‐pulse measurements at 30°C demonstrate RT switching with *E*_c_ = 54 Vμm^−1^ and *P*_s_ = 0.92 μCcm^−2^ (Figure 25d). Furthermore, an open‐circuit voltage signal of ∼23 mV remains unchanged even after 8 months (Figure 25d), directly evidencing long‐term polarization retention at RT. While the relatively high *E*_*c*_ and long pulse widths indicate that further optimization is needed for device integration, the study establishes that both RT ferroelectric switching and negligible polarization decay can be simultaneously achieved in triazole‐engineered helical columnar systems.

## 7 Summary and Outlook

FCLCs represent a compelling organic‐memory concept because an axially polarized, bistable state can, in principle, be written and erased within nanoscale columnar domains. Yet the central bottleneck remains that most electrically responsive CLCs show little macroscopic remanence at zero bias, relaxing into paraelectric/antiferroelectric states. In this Concept, “genuine” FCLC performance is therefore framed as the simultaneous fulfillment of three criteria: formation of axial polarization, reversible switching, and robust polarization retention in the zero‐field state. A key mechanistic insight is that polarization retention is governed by whether rotation‐driven depolarization pathways are kinetically blocked. Directional H‐bonding and steric confinement can immobilize polar motifs, acting as “supramolecular locking,” while the formation of well‐defined intracolumnar order—often manifested as helical column architectures—provides a practical structural handle for diagnosing and engineering long‐lived retention.

Across amide‐derived systems, early work largely targeted E‐field alignment (the “amide handle”), while later fan‐shaped designs clarified how adding free volume and reorganizing core–shell packing could unlock cooperative switching and measurable retention, albeit typically at elevated temperatures and/or with slow kinetics. Core expansion (e.g., benzotrithiophene‐based discotic LC) further highlighted that improved long‐range order and reduced defect density could enhance retention, even when *E*_c_ remained relatively high.

BTA platforms offered especially instructive structure–property lessons: linear‐chain BTAs could switch but depolarized rapidly at high temperature, whereas chain shortening increased packing density and remnant polarization and enabled long retention at RT; branched chains dramatically improved retention by promoting more ordered plastic‐crystalline assemblies but at the cost of large *E*_*c*_ values. Notably, blending linear/branched BTAs provided a practical compromise between retention and operating voltage and converting amide to thioamide increased dipole strength while reducing the *E*_c_—showing that retention and drive voltage could be decoupled.

Urea‐core systems showed that intentionally forming helical structures (e.g., by introducing chiral chains) was an effective way to increase the switching barrier and stabilize macroscopic polarization. This design concept ultimately led to rationally redesigned polycatenar molecules that could switch at RT while maintaining long‐lived polarization.

Finally, 1,2,3‐triazole linkages—combining a large dipole moment with H‐bonding capability—have recently advanced from E‐field alignment control to true RT‐operable FCLC behavior when bulky triazole motifs stabilized an asymmetric helical columnar phase and suppressed post‐switch relaxation.

Looking forward, achieving materials that meet device‐relevant metrics will likely require simultaneous optimization of (i) supramolecular locking strength (i.e., polarization retention), (ii) the viscosity/defect landscape (i.e., alignment quality and switching speed), and (iii) *E*_*c*_ and pulse‐width requirements (i.e., power consumption and device integration). In particular, to enable applications such as FeRAM, the polarization switching speed and lowering *E*_c_ are required to be improved. To move toward these goals, future FCLC design should aim to maintain helical order while reducing intermolecular interactions. For example, viable strategies may include lowering the molecular weight of FCLC mesogens or intentionally employing weaker intermolecular interactions to facilitate faster switching dynamics, while preserving helical columnar order. Notably, if FCLC behavior can be realized by relying on non‐H‐bonding constraints, such as steric hindrance, rather than the strong H‐bonding commonly used in current FCLC systems, this could open a new pathway for next‐generation FCLCs with improved switching performance.

An equally important but still underexplored issue is fatigue, or endurance under repeated polarization switching. To date, systematic endurance studies on FCLCs remain scarce. This is not because fatigue is a secondary concern, but rather because the field is still at a stage where only a limited number of systems satisfy the more fundamental requirements of genuine ferroelectricity—namely, axial polarization, reversible switching, and retention at zero field—with sufficient reproducibility. In many reported FCLC systems, switching still requires relatively high *E*_c_, long pulse widths, elevated temperatures, or highly optimized alignment conditions, while the measured response can remain sensitive to defect reorganization, ionic contributions, and sample‐to‐sample variability. Under such circumstances, it is difficult to distinguish intrinsic polarization fatigue from extrinsic degradation processes such as loss of alignment, gradual structural relaxation, or charge‐injection artifacts. Nevertheless, for FCLCs to progress toward practical nonvolatile memory applications, endurance must ultimately be established as a key benchmark. Accordingly, fatigue resistance should now be recognized as both a current limitation of the field and a critical target for future work, requiring standardized cycling protocols and post‐cycling structural/electrical analyses that can clarify how repeated switching perturbs supramolecular locking and long‐range columnar order.

With such combined molecular–device engineering, FCLCs can move from rare proof‐of‐principle systems toward practical, nonvolatile, ultra‐high‐density organic memory elements.

## Conflicts of Interest

The authors declare no conflicts of interest.

## Data Availability

Data sharing not applicable to this article as no datasets were generated or analyzed during the current study.
